# Morphological and immunophenotypic characterization of perivascular interstitial cells in human glioma: Telocytes, pericytes, and mixed immunophenotypes

**DOI:** 10.18632/oncotarget.27340

**Published:** 2020-01-28

**Authors:** Lubov Mitrofanova, Anton Hazratov, Boris Galkovsky, Andrey Gorshkov, Danila Bobkov, Dmitry Gulyaev, Evgeny Shlyakhto

**Affiliations:** ^1^Almazov National Medical Research Centre, Pathomorphology Research Laboratory, St. Petersburg, Russia; ^2^Smorodintsev Research Institute of Influenza, Laboratory of Intracellular Signaling and Transport, St. Petersburg, Russia; ^3^Institute of Cytology of the Russian Academy of Science, Laboratory of Cell Biology in Culture, St. Petersburg, Russia; ^4^Almazov National Medical Research Centre, Research Department of Neurosurgery, St. Petersburg, Russia; ^5^Almazov National Medical Research Centre, General Director, St. Petersburg, Russia

**Keywords:** telocytes, pericytes, confocal microscopy, primary culture of glioma telocytes, glioblastoma

## Abstract

Telocytes (Tcs) and pericytes (Pcs) are two types of perivascular interstitial cell known to be widespread in various organs and tissues, including the brain. We postulated that Tcs and Pcs may be involved in glioblastoma (GBM) neovascularization.

**Objective:**

Morphological study of Tc and Pc roles in GBM.

**Materials and Methods:**

Samples from 15 GBM, 10 diffuse astrocytoma, as well as 5 control samples were studied. We used immunohistochemistry (IHC) with antibodies (Abs) to GFAP, Ki-67, CD117, NeuroD1, NG2, CD34, and SMA. Confocal laser scanning microscopy (CLSM) of 4 glioma tissue cultures and 4 GBM sections was performed with GFAP, CD117, CD34/connexin43, NeuroD1/connexin43, CD34/NG2 and CD13/CD117 Abs. Electron microscopy (EM) of GBM was performed in 4 cases.

**Results:**

The presence of Tcs and Pcs was shown in GBM (IHC, EM, CLSM) and glioma cultures (CLSM). The Tc immunophenotype was CD117^+^/CD34^+^/connexin43^+^/NeuroD1^+^. The Pc immunophenotype was SMA^+^/NG2^+^/CD13^+^. The number of Tcs in GBM specimens was 10 times higher than in astrocytoma. We also identified CD13/CD117 and CD34/NG2 co-expressing cells in GBM blood vessels.

**Conclusion:**

Four immunophenotypes were found in GBM vessels, corresponding to endotheliocytes, Pcs, Tcs, and a mixed Pc/Tc immunophenotype. These and forthcoming improvements in our understanding of the origin and function of Tcs, including their relationship with Pcs, are necessary steps in oncology. Study of these cell types (Tcs, Pcs) and their roles in brain tumor oncogenesis will likely enable improved targeted therapies and support development of new forms of anti-neoplastic drugs.

## INTRODUCTION

In 1893, Spanish neuroscientist Santiago Ramón-y-Cajal described cells located in the muscle wall of the gastrointestinal tract which are special elements of the intramural nerve plexus and which regulate gastrointestinal motility; he termed them “interstitial neurons”. Later (1977–1982), M.S. Faussone-Pellegrini and L. Thuneberg, using electron microscopy data and independent of each other, came to the conclusion that the so-called “interstitial neurons” are not related to nervous tissue, but rather are derived from mesenchyme [1, 2]. In 2010, Popescu and Faussone-Pellegrini termed these cells telocytes (Tcs) [3]. Tcs are a unique type of interstitial cells with specific processes (telopodia) and their dilated segments (podoms) [4]. Tcs simultaneously belong to interstitial, endothelial, smooth muscle, nerve, mast, and hematopoietic stem cell immunophenotypes. They express CD117, vimentin, CD34, SMA, S100, NSE, as well as the gap junction protein connexin-43 [5, 6, 7]. Tcs form a 3D network in almost all organs, and they are involved in inflammation, regeneration, and angiogenesis; through multiple diverse cell-to-cell contacts and micro-vesicle involvement, they can coordinate inter-cellular interactions [8, 9, 10, 11]. They have pacemaker activity and express inflammatory mediators and angiogenesis factors, such as PDGFR, VEGF, EGF, FGF, and TGF [12, 13]. Popescu et al. [14] proved the presence of Tcs in the dura mater, the choroid plexus of the ventricles, and in the subventricular zone of rat brain. Tcs establish close contacts with blood capillaries, nerve fibers, and stem cells. Neural stem cells can participate in adult neurogenesis. Xu et al. [15] found Tcs in canine dura mater, and Contarero et al. [16] showed that they are in close contact with the vessels of the microvasculature. Zheng et al. [17, 18] have demonstrated the involvement of interstitial pacemaker cells at the air-blood barrier in lung vessels; they have also demonstrated their presence between smooth muscle cells and endothelial cells of lung capillaries. The involvement of Tcs in post-myocardial-infarction angiogenesis has been shown by Manole et al. [19].

Glioblastoma (GBM) is the most common malignant primary brain tumor, making up 54% of all gliomas and 16% of all primary brain tumors [20]. GBM remains an incurable tumor with a median survival of only 15 months [21]. Recently, special attention has been paid to the role of tumors’ stroma in carcinogenesis, which is logical given that some targeted therapies are directed at it. In particular, avastin (bevacizumab), a drug that suppresses tumor neovascularization, has shown promising results. This is especially relevant to GBM, in which the formation of atypical (aberrant) and poorly functioning vessels, leading to hypoxia, tumor necrosis, and low efficiency of drug delivery, have been shown [22].

At least five mechanisms by which gliomas achieve neovascularization have been described: vascular co-option; angiogenesis; vasculogenesis; vascular mimicry; and (most recently) GBM-endothelial cell transdifferentiation [23]. Vascular co-option is the first mechanism by which gliomas achieve their vasculature. This process involves organization of tumor cells into cuffs around normal microvessels [24]. Vascular co-option is followed by the development of new vessels from pre-existing ones, known as angiogenesis [25]. Glioma-associated sprouting angiogenesis starts with an angiopoietin-mediated breakdown of existing vessels. After vascular co-option, persistent up-regulation of ANG-2 and TIE-2 in endothelial and tumor cells promotes disruption of endothelial and perivascular cell junctions, resulting in vessel disruption. In the presence of ANG-2, VEGF promotes migration and proliferation of endothelial cells and stimulates sprouting of new blood vessels. The end result of the neoplastic angiogenic process is a characteristically abnormal vascular network featuring dilated and tortuous vessels, abnormal branching, and arteriovenous shunts which may lead to abnormal perfusion. A third mechanism of tumor neovascularization, vasculogenesis, involves differentiation of circulating bone marrow-derived cells known as endothelial progenitor cells [26]. The fourth mechanism of glioma vascularization, vascular mimicry, is defined as the ability of tumor cells to form functional vascular networks [27]. The fifth described mechanism of glioma neovascularization involves the transdifferentiation of glioma cells into an endothelial phenotype.

GBM stem cells are a source of pro-angiogenic factors, such as VEGF. They are located in the perivascular niche of the tumor microenvironment and may be sensitive to therapies targeting tumor vasculature [28]. Mou et al. [29] showed that Tcs and other breast cancer stromal cells contribute to the formation of the typical tumor structure, promote the proliferation of tumor cells, and suppress their apoptosis *in vitro*. Mirancea et al. [30] found that Tcs are a component of tumor stroma in basal cell carcinoma and squamous cell carcinoma. Tcs can be found in specialized somatic synapses forming a 3D network inside peritumoral stroma. It is the authors’ view that Tcs likely promote the invasive behavior of microtumors.

Pcs were first discovered in 1873 by the French scientist Charles-Marie Benjamin Rouget and were originally called Rouget cells [31]. They were renamed a few years later due to their localization and close contact with endothelial cells. In the brain, Pcs are believed to be located in precapillary arterioles, capillaries, and postcapillary venules. Pcs of the central nervous system are normally located on the outer surface of microvessels and share a common basement membrane with endothelial cells. A pericyte’s cytoplasmic processes may be in contact with several endothelial cells simultaneously, and various types of structure are possible, depending on the vessel size.

The highest described Pc densities have been specific to the central nervous system [32, 33]. The Pc immunophenotype is: NG2^+^, PDGFRb^+^, CD13^+^, αSMA^+^, CD146^+^, desmin^+^, vimentin^+^, and K_ir_6.1 potassium channel complex^+^ [51, 52, 53, 54, 55]. Sun et al. also describe α-SMA-expressing Pcs in GBM vessels [56].

Pcs have contact with glia (astrocytes) and are involved in inflammation [36]. They are known to regulate blood flow, blood-brain barrier permeability [34, 35], tissue homeostasis, and regeneration in vascular and other tissues. Pcs also have stem cell function, including the ability to differentiate into: adipocytes; chondrocytes; osteoblasts; fibroblasts; mesenchymal stem cells (MSC) [37]; vascular cells; oligodendrocytes and astrocytes [38, 39]; as well as neuronal cells of the central nervous system [40, 41, 42]. Importantly, all of these functions, including stem cell function, are also attributed to Tcs [43].

Today, in addition to known types (astrocytes, oligodendrocytes, microglia, ependymocytes), a fifth type of glia (NG2^+^ synantocytes) has been identified in the central nervous system [44]. Unlike Tcs and Pcs, synantocytes do not express GFAP. It is believed that synantocytes are components of synapses and that they are involved in stabilization of neuronal cytoskeleton and control of myelin integrity. They are thought to be responsible for damage to nerve fibers and formation of glial scars. NG2^+^ glial cells have been proven to support neuron function and survival through control of the neuroimmunological system [45]. They are also considered to be precursors of oligodendrocytes. An important and functionally significant feature of NG2-glia is their presence throughout the brain, both in gray and white matter, throughout postnatal development and in adulthood [46].

Like Tcs, NG2-glia are also present in neurogenic niches, including the subventricular zone (SVZ) and the dentate gyrus of the hippocampus [47, 48]. It is well known that, in the postnatal and mature brain, NG2-glia contain the largest population of endogenous/resident progenitor cells (4 - 8% of total cells, depending on the area of the brain), can quickly “respond” to any type of injury, and have high potential for re-population of lesions [49].

Finally, interactions between NG2-glia and other types of nerve cells can vary in different areas of the brain [50]. Despite the aforementioned research work, the exact relationship between Tcs and NG2-glia is unclear at the moment. Given the presence of Tcs in various organs (including the brain), their participation in angiogenesis, and their presence in some tumors, we hypothesized that Tcs may also be involved in neovascularization by GBM.

### Objective

morphological study of perivascular interstitial cells (Tcs and Pcs) in GBM.

## RESULTS

### Histological and immunohistochemical study

**Figure 1:**
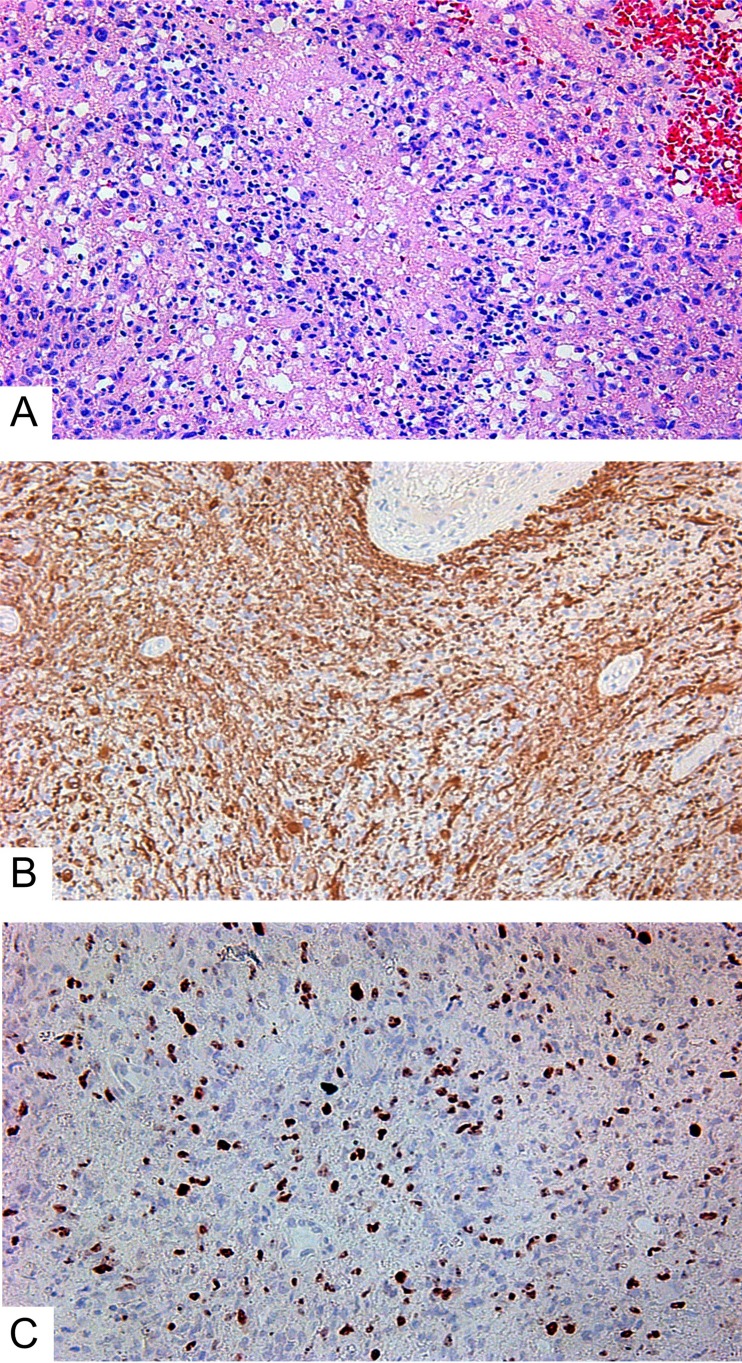
Small cell glioblastoma. **(A)** Hematoxylin and eosin; **(B)** GFAP expression in glioblastoma’s cells; **(C)** Ki-67 expression in glioblastoma. All slides at 200×.

We investigated 15 GBM, and of them: 2 were giant cell GBMs; 3 were GBMs with a primitive neuronal component; and 10 were small cell GBMs (Figure 1A). Two patients underwent additional surgery due to tumor recurrence following their initial treatments (resection, chemotherapy, radiation therapy). Fourteen GBMs were primary. One was secondary; the patient underwent treatment (surgery, chemotherapy, radiation therapy) for hemistocytic astrocytoma 4 years earlier.

**Figure 2:**
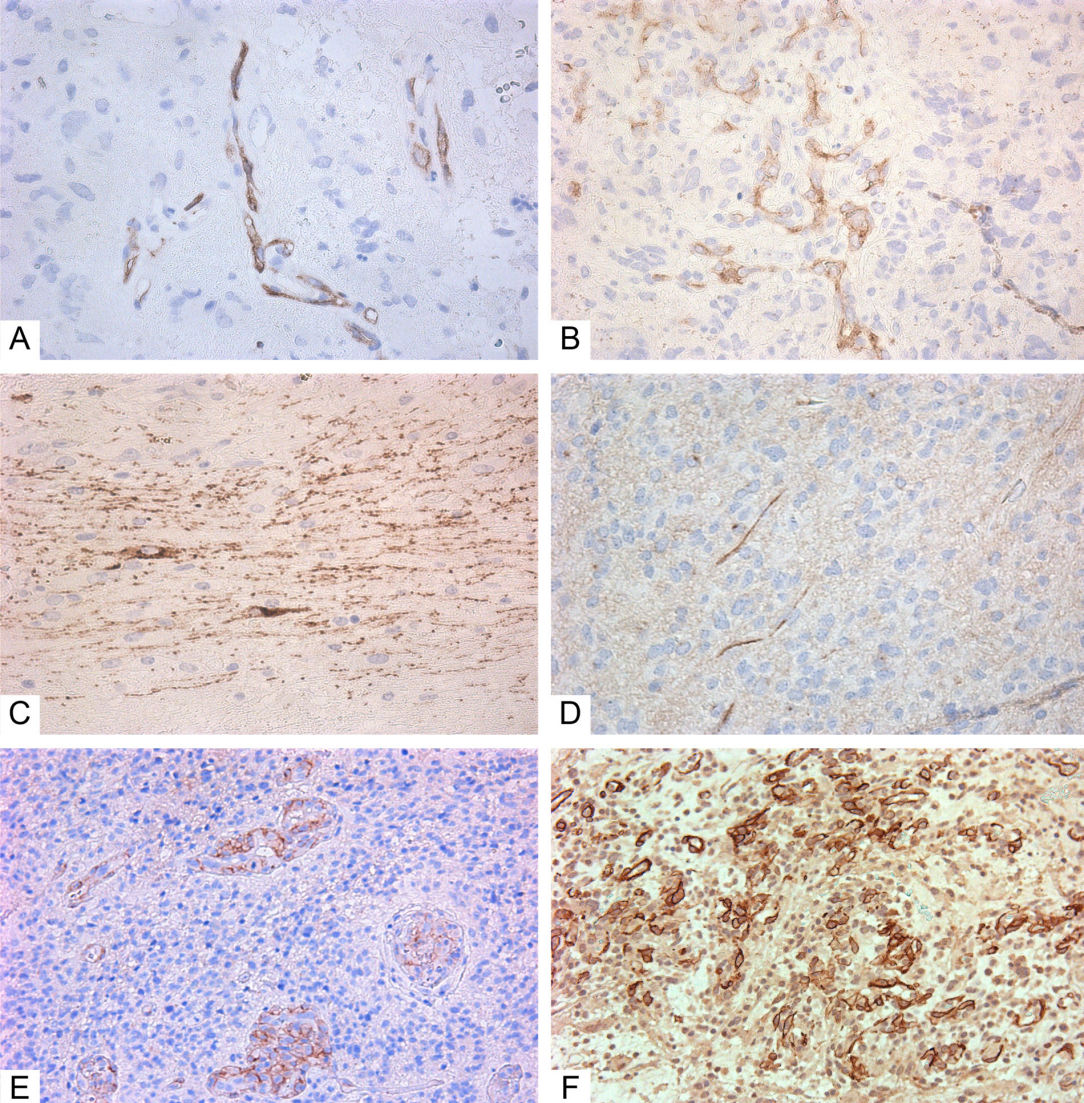
Immunohistochemistry of glioblastoma. **(A–D)** Giant cell glioblastoma; **(E–F)** Small cell glioblastoma). **(A)** CD117+ cells in vessels (Genemed Ab); **(B)** CD117+ cells in vessels (Diagnostic BioSystems Ab); **(C)** CD117+ cells in glial scar (Genemed Ab); **(D)** CD117+ (Diagnostic BioSystems Ab) cells among tumor cells; **(E)** NG2+ cells in vessels; **(F)** NG2+ cells among tumor cells and in vessels. Antibodies are detailed in the Supplementary Materials. All slides at 200×. CD117+ cells and NG2+ cells are stained brown.

According to the IHC data, all GBMs expressed GFAP (Figure 1B). Ki-67 indexes were from 21 to 50%, with an average of 27.3 ± 1.9% (Figure 1C). Expression of the Tc marker CD117 was observed in the vascular walls (Figure 2A and 2B), in the glial scar (Figure 2C), and (less often) on the periphery of tumor cells like a braid (Figure 2D). NG2 was also expressed in those locations (Figure 2E and 2F).

**Figure 3:**
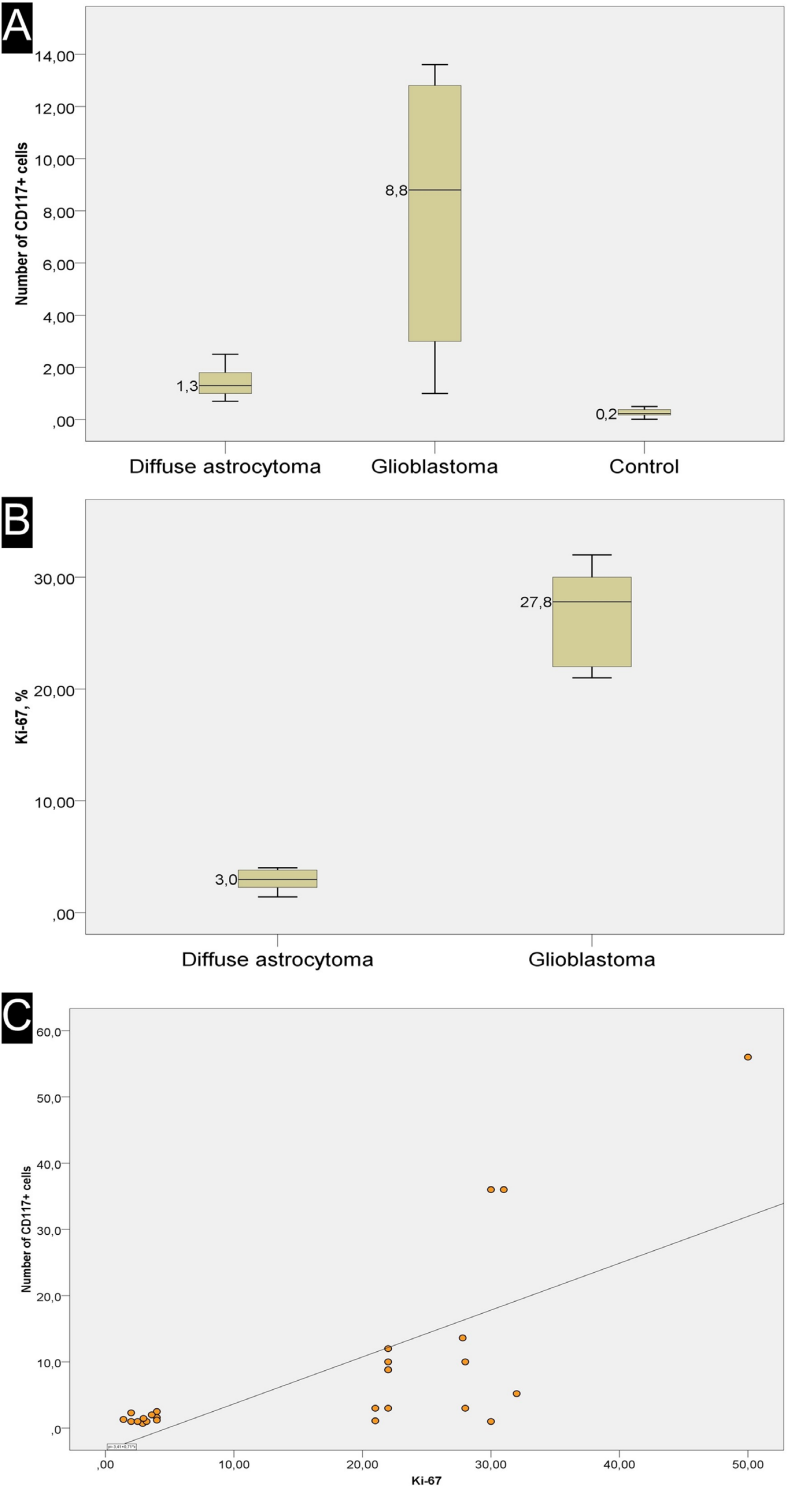
Overview of Ki-67 expression and correlation with number of CD117^+^ cells. **(A)** the average number of CD117+ cells in one ^*^field of view. **(B)** the average number of Ki-67+ cells in one ^*^field of view. **(C)** scatter plot with a regression curve showing the linear relationship between Ki-67 index and the number of telocytes. ^*^(400×)

**Figure 4:**
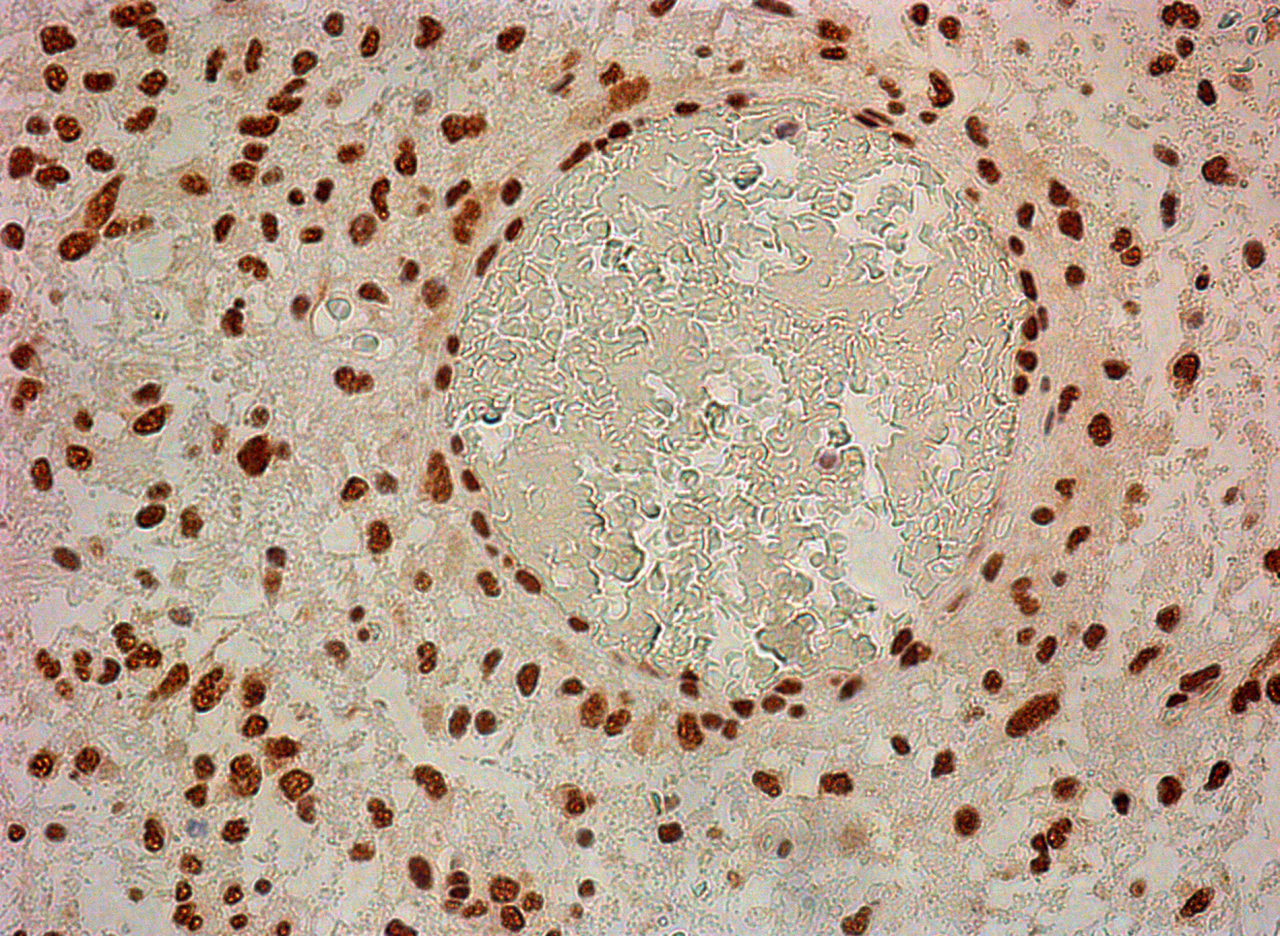
Neuro D1 in tumor cell nuclei and in vessel cells. 200×.

Counting of CD117-expressing cells showed that the average number of cells with expression of this antigen ranged from 1 to 56%, with the latter (high) value seen in a post-operative glial scar (56%). In the GBM group, the average number of CD117^+^ cells was 13.8 ± 4.1 % (Figure 3A). NeuroD1 expression was observed not only in the vast majority of tumor cells, but also in vascular cell nuclei (Figure 4).

**Figure 5:**
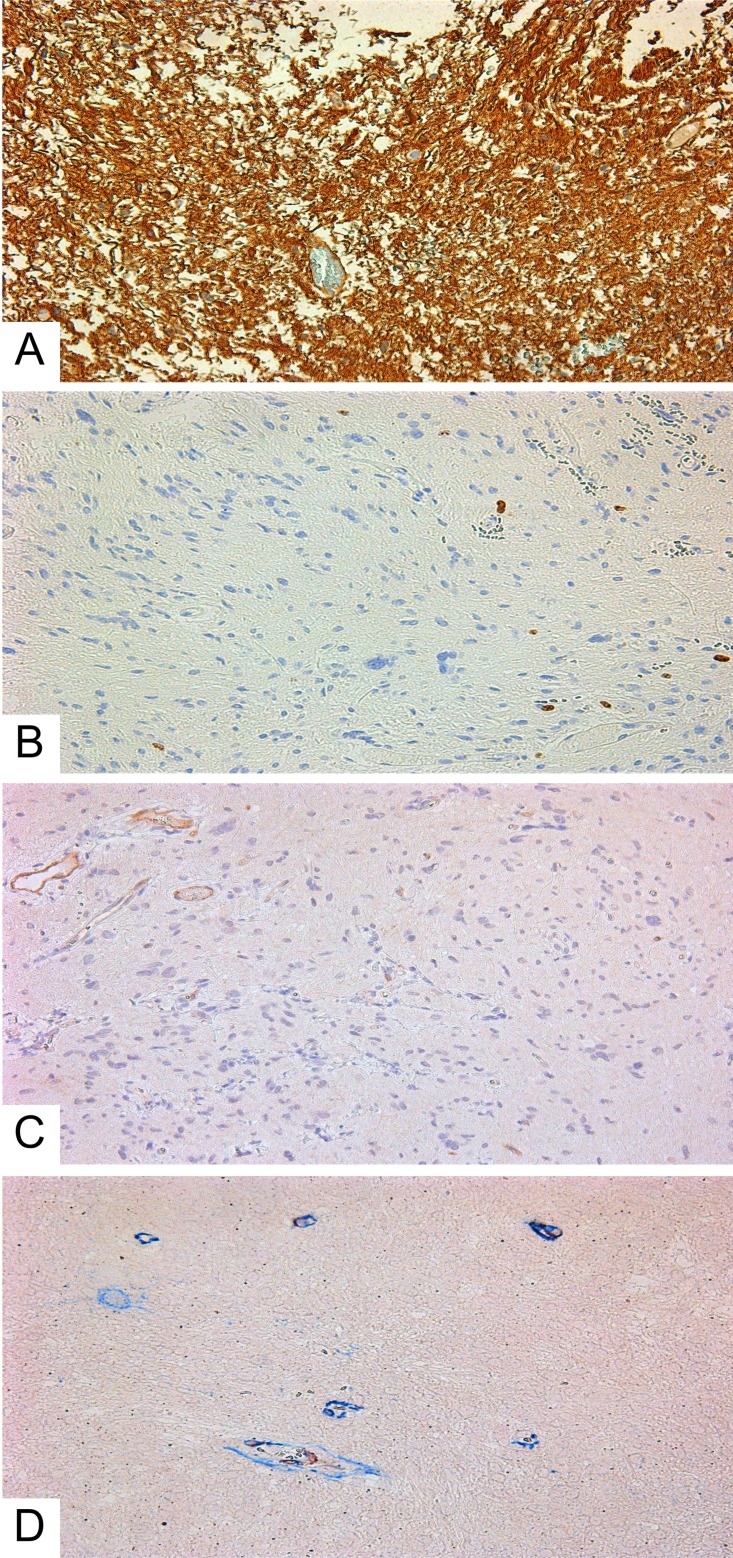
Diffuse astrocytoma. **(A)** GFAP expression in tumor cells; **(B)** Ki-67 expression in astrocytoma’s cells; **(C)** CD117+ cells in diffuse astrocytoma vessels. **(D)** Double-stain IHC of diffuse astrocytoma with CD34/CD117 antibodies CD117: red; CD34: blue; co-expression: maroon; All slides at 200×.

Diffuse astrocytomas (comparison group) also expressed GFAP; their Ki-67 index was from 1.4 to 4, with 2.96 ± 0.30% on average (Figure 5A, 5B, and 3B). In astrocytomas, CD117-expressing cells with Tc characteristics were extremely rarely seen in vessel walls (Figure 5C and 5D). The average number of CD117-expressing cells in the astrocytoma group was 1.46 ± 0.18, significantly less than in GBMs (Figure 3A). Correlation analysis revealed a significant association between Ki-67 and the number of CD117^+^ Tcs. In addition, a linear correlation between tumor cell proliferative activity index and number of Tcs was seen (Figure 3C).

**Figure 6:**
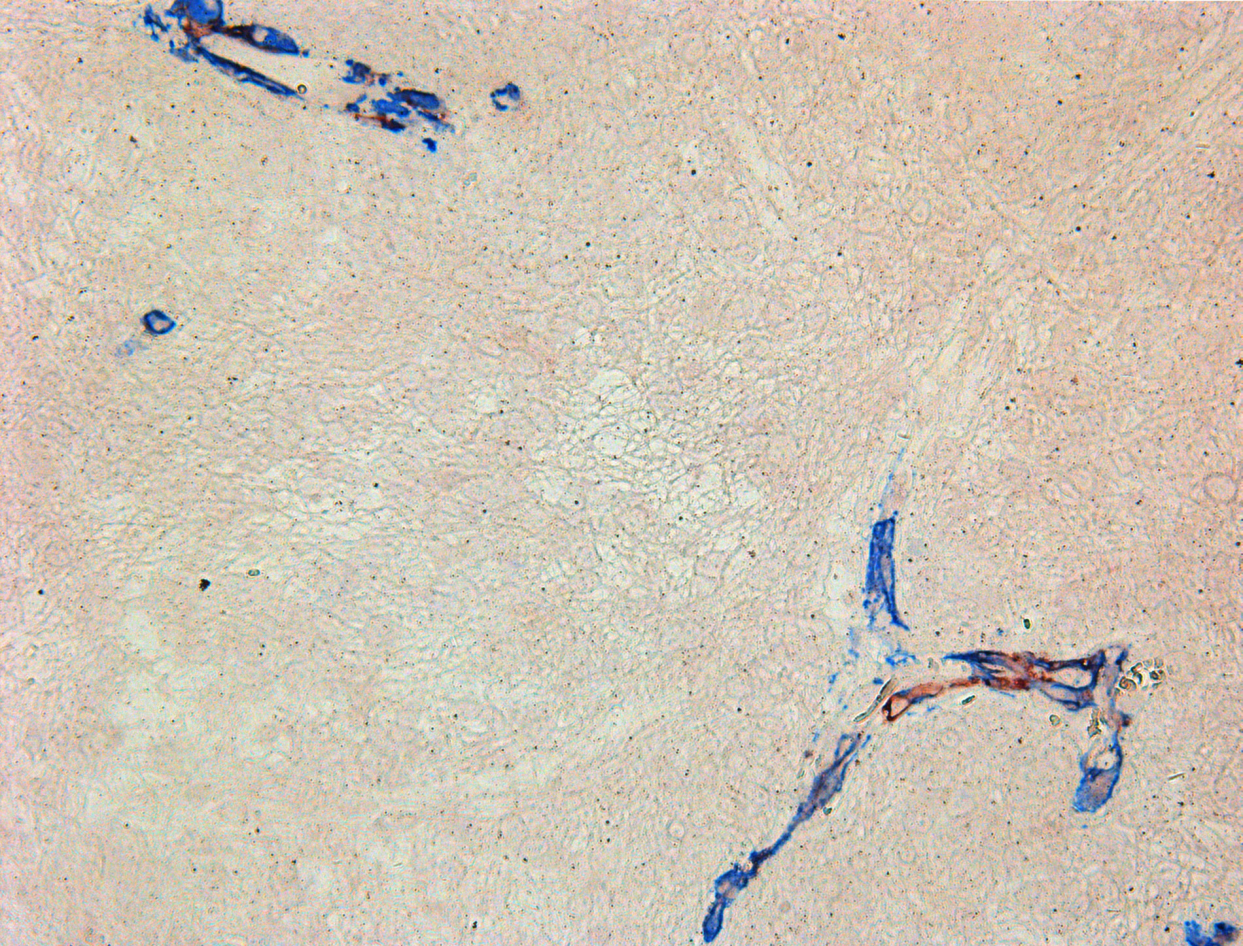
Double-stain IHC of small cell glioblastoma with NG2/SMA antibodies. NG2: red; SMA: blue; co-expression: maroon; 200×.

Glioblastoma IHC staining with NG2/SMA antibody cocktail demonstrated co-expression in vascular wall cells (Figure 6). This fact was interpreted as an indication of Pc presence in tumor vessels.

**Figure 7:**
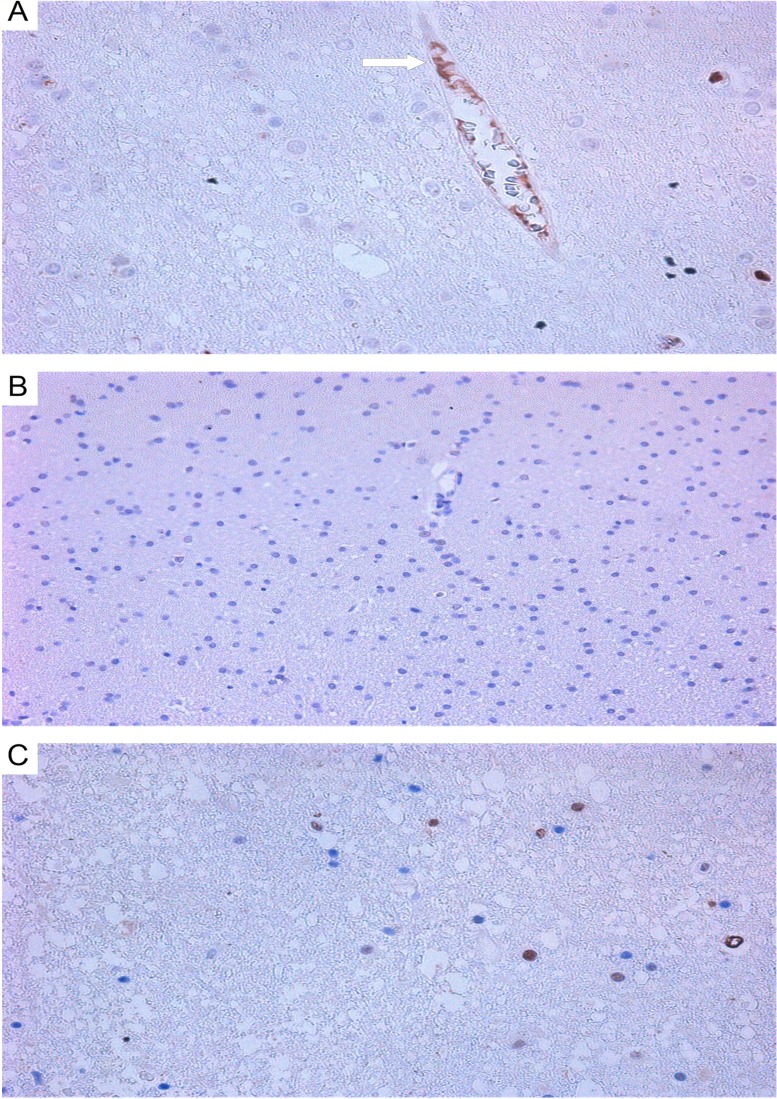
White matter of the normal brain. **(A)** CD117+ cells in the vascular wall (white arrow); **(B)** lack of NG2 expression on the cells of vascular walls; **(C)** expression of NeuroD1 on oligodendrocyte nuclei; All slides at 200×.

For comparison with tumor tissue, we also performed an IHC study of frontal lobes from patients who died from cardiovascular disease. CD117^+^ cells were detected extremely rarely in vessel walls (Figure 7A) and somewhat more in white matter, up to 0.5%, with an average of 0.26±0.09% (see Table 1). NG2^+^ cells were seen even more rarely. In none of the 5 cases did we see NG2 expression on vascular wall cells (Figure 7B). NeuroD1 expression varied from patient to patient, with the number of expressing cells ranging from 30-87%. Expression was in the nuclei of neurons and glial cells (white and grey matter) (Figure 7C).

**Table 1:** Overview of patients, specimens, and analytical methods

№	Sex	Age	^*^Type of glioma disease (autopsy) Ki-67 (%), CD117^+^ cells (%)	Tumor location and dimensions (MRI)	Cause of death (in control group)	Methods
**Glioblastomas**						
**1**	**F**	**73**	**Secondary giant cell glioblastoma, Grade IV, IDH-mutant, Ki-67 – 22%, CD117^+^ cells – 8.8%**	**Left frontal and temporal lobes, at a depth of 5mm, 35x35x34mm**	**-**	**H, IHC, MA**
**2**	**F**	**48**	**small cell glioblastoma, Grade IV, IDH-wildtype, Ki-67 – 30%, CD117^+^ cells – 36.0%**	**Right frontal and parietal lobes, 55x42x39mm**	**-**	**H, IHC, MA**
**3**	**F**	**59**	**Giant cell glioblastoma with primitive neuronal component, Grade IV, IDH-wildtype, Ki-67 – 21%, CD117^+^ cells – 1.0%**	**Left parietal lobe, 17x13x17mm**	**-**	**H, IHC, MA**
**4**	**F**	**60**	**glioblastomas with primitive neuronal component, Grade IV, IDH-wildtype, Ki-67 – 50%, CD117^+^ cells – 1.1%**	**Left temporal lobe, 64x40x56mm**	**-**	**H, IHC, MA, CLSM**
**5**	**F**	**64**	**glioblastomas with primitive neuronal component, Grade IV, IDH-wildtype, Ki-67 – 32%, CD117^+^ cells – 2.5%**	**Right temporal lobe, 33x32x30mm**	**-**	**H, IHC, MA, CLSM**
**6**	**F**	**73**	**small cell glioblastoma, Grade IV, IDH-wildtype, Ki-67 – 30%, CD117^+^ cells – 5.2%**	**Right temporal lobe, at a depth of 1cm,36x35x44mm**	**-**	**H, IHC, MA, DSIHC, CLSM**
**7**	**F**	**61**	**small cell glioblastoma, Grade IV, IDH-wildtype, Ki-67 – 22%, CD117^+^ cells – 3.0%**	**Right parietal and occipital lobes, thalamus and basal nuclei, 36x36x39mm**	**-**	**H, IHC, MA, DSIHC, CLSM**
**8**	**F**	**63**	**small cell glioblastoma, Grade IV, IDH-wildtype, Ki-67 – 21%, CD117^+^ cells – 2.5%, CD117^+^ cells – 10.0%**	**Right frontal and parietal lobes, 37x30x29mm**	**-**	**H, IHC, MA, DSIHC**
**9**	**F**	**61**	**small cell glioblastoma, Grade IV, IDH-wildtype, Ki-67 – 28%, CD117^+^ cells – 56.0%**	**Right frontal lobe, thalamus, 45x72x54mm**	**-**	**H, IHC, MA, DSIHC**
**10**	**F**	**61**	**small cell glioblastoma, Grade IV, IDH-wildtype, Ki-67 – 22%, CD117^+^ cells – 12.0%**	**Right frontal and temporal lobes, 31x17x34mm**	**-**	**H, IHC, MA, DSIHC**
**11**	**F**	**54**	**small cell glioblastoma, Grade IV, IDH-wildtype, Ki-67 – 22%, CD117^+^ cells – 13.6%**	**Right frontal and temporal lobes, 47x28x20mm**	**-**	**H, IHC, DSIHC, MA**
**12**	**F**	**60**	**small cell glioblastoma, Grade IV, IDH-wildtype, Ki-67 – 28%, CD117^+^ cells – 3.0%**	**Left frontal and parietal lobes, 40x31x34mm**	**-**	**H, IHC, PC, CLSM, EM, MA**
**13**	**M**	**62**	**small cell glioblastoma, Grade IV, IDH-wildtype, Ki-67 – 22%, CD117^+^ cells – 8.8%**	**Right parietal lobe, 26x15x19mm**	**-**	**H, IHC, PC, CLSM, EM, MA**
**14**	**M**	**56**	**small cell glioblastoma, Grade IV, IDH-wildtype, Ki-67 – 31%, CD117^+^ cells – 36.0%**	**Right frontal and parietal lobes, 63x54x50mm**	**-**	**H, IHC, EM, MA**
**15**	**F**	**76**	**small cell glioblastoma, Grade IV, IDH-wildtype, Ki-67 – 22%, CD117^+^ cells – 10.0%**	**Right and left frontal lobes and corpus callosum, 65x54x52mm**	**-**	**H, IHC, EM, MA**
**Astrocytomas**						
**16**	**F**	**71**	**diffuse astrocytomas, Grade II, NOS categories, Ki-67 – 2%, CD117^+^ cells – 1%**	**Left temporal lobe, 45x20x10mm**	**-**	**H, IHC, MA, DSIHC, PC, CLSM**
**17**	**M**	**47**	**diffuse astrocytomas, Grade II, NOS categories, Ki-67 – 4%, CD117^+^ cells – 2.5%**	**Right frontal and parietal lobes, 44x26x24mm**	**-**	**H, IHC, MA, DSIHC, PC, CLSM**
**18**	**M**	**57**	**diffuse astrocytomas, Grade II, NOS categories, Ki-67 – 4%, CD117^+^ cells – 1.6%**	**Left parietal and occipital lobes, corpus callosum, 84x66x11mm**	**-**	**H, IHC, MA**
**19**	**F**	**40**	**diffuse astrocytomas, Grade II, NOS categories, Ki-67 – 4% CD117^+^ cells – 0.7%**	**Right parietal lobe, thalamus, 33x30x30mm**	**-**	**H, IHC, MA**
**20**	**F**	**61**	**diffuse astrocytomas, Grade II, NOS categories, Ki-67 – 1,4%, CD117^+^ cells – 2.3%**	**Left frontal and parietal lobes, corpus callosum, 60x60x60mm**	**-**	**H, IHC, MA**
**21**	**M**	**59**	**diffuse astrocytomas, Grade II, NOS categories, Ki-67 – 2.5%, CD117^+^ cells – 1.3%**	**Left temporal lobe, 33x33x32mm**	**-**	**H, IHC, MA**
**22**	**M**	**71**	**diffuse astrocytomas, Grade II, NOS categories, Ki-67 – 4%, CD117^+^ cells – 1.2%**	**Right frontal lobe, 60x55x61mm**	**-**	**H, IHC, MA**
**23**	**F**	**35**	**diffuse astrocytomas, Grade II, NOS categories, Ki-67 – 3.6%, CD117^+^ cells – 1.0%**	**Right parietal lobe, 21x22x21mm**	**-**	**H, IHC, MA**
**24**	**F**	**39**	**diffuse astrocytomas, Grade II, NOS categories, Ki-67 – 2.9%, CD117^+^ cells – 2.0%**	**Left temporal lobe, 18x32x18 mm**	**-**	**H, IHC, MA**
**25**	**F**	**60**	**diffuse astrocytomas, Grade II, NOS categories, Ki-67 – 3.2%, CD117^+^ cells – 1.0%**	**Left temporal lobe, 10×11×12 mm**	**-**	**H, IHC, MA**
**Normal brain**						
**26**	**M**	**68**	**Coronary artery disease, CD117+ cells –0.01%**	**no tumor**	**Pulmonary embolism**	**H, IHC, MA**
**27**	**M**	**40**	**Coronary artery disease, CD117+cells –0.17%**	**no tumor**	**Heart failure**	**H, IHC, MA**
**28**	**M**	**53**	**Coronary artery disease, CD117+ cells –0.38%**	**no tumor**	**Pulmonary embolism**	**H, IHC, MA**
**29**	**F**	**51**	**Coronary artery disease, CD117+ cells –0.23%**	**no tumor**	**Myocardial infarction**	**H, IHC, MA**
**30**	**F**	**61**	**Coronary artery disease, CD117+ cells- 0.5%**	**no tumor**	**Myocardial infarction**	**H, IHC, MA**

*2016 World Health Organization Classification of Tumors of the Central Nervous System [57]. Abbreviations: H, histology; PC, primary culture of telocytes from gliomas; IHC, immunohistochemistry; MA, morphometric analysis; DSIHC, double stain immunohistochemistry; CLSM, confocal laser scanning microscopy; EM, electron microscopy; MRI, magnetic resonance imaging; NOS categories, not otherwise specified (tumor samples were not tested for IDH gene mutations).

### Primary culture

**Figure 8:**
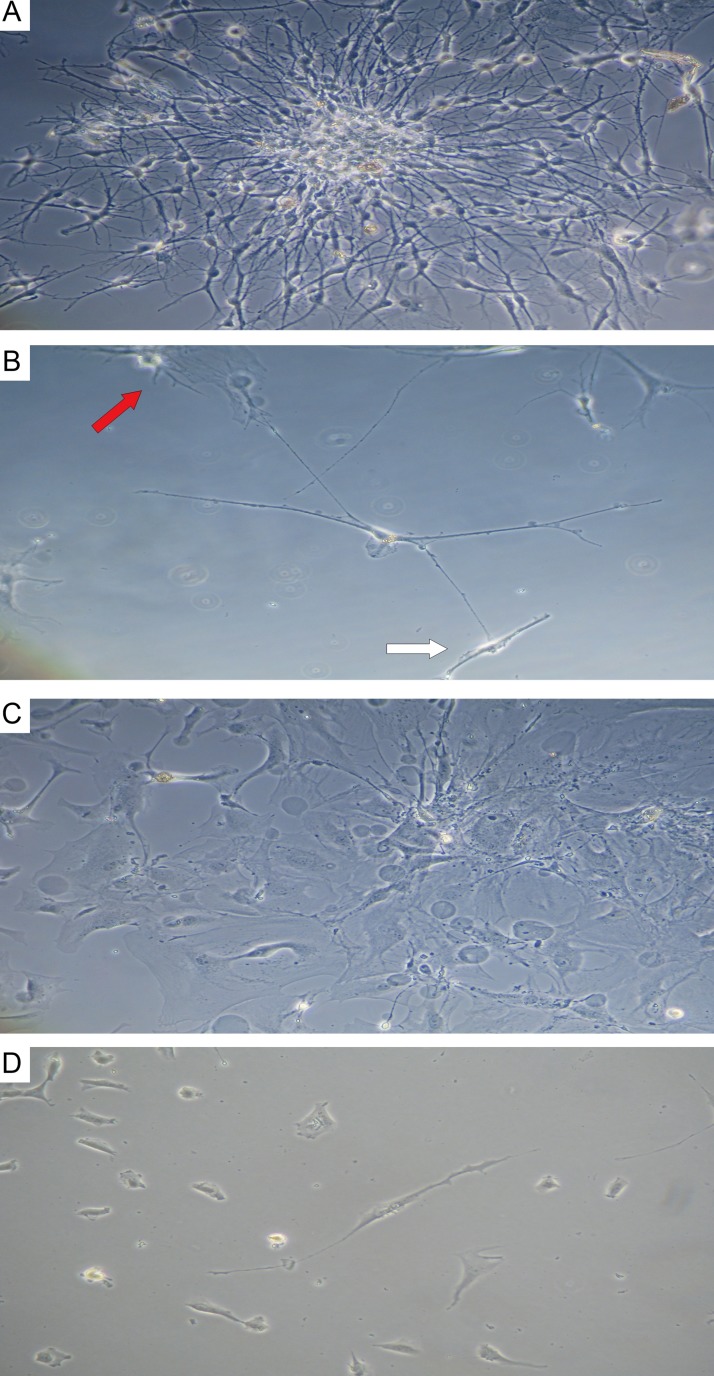
Astrocytoma and glioblastoma primary culture at 7 days. **(A)** stellate cells in astrocytoma colony; **(B)** telocyte (center) featuring a small, ovoid body and 4 telopods in contact with a fibroblast-like cell (white arrow) and a tumor cell (red arrow); phase contrast microscopy at 200×. **(C)** stellate cells in glioblastoma colony; **(D)** telocyte (center) featuring a small, ovoid body and 2 telopods; 400×.

In primary cultures of GBM and astrocytoma, typical Tcs were detected (Figure 8). They feature a fusiform cell body and two long prolongations. The morphology of Tcs *in vitro* is different from that seen in tumor cells. After 7 days of culture, typical Tc morphological features appeared (seen under light microscopy): small, oval-shaped cell bodies with extremely long, thin, moniliform prolongations (telopodes) extending from cell bodies. In primary cultures, Tcs often were seen mixed with tumor cells; Tc telopodes typically can be seen extending directly into contact with tumor cells.

### Confocal laser scanning microscopy

**Figure 9:**
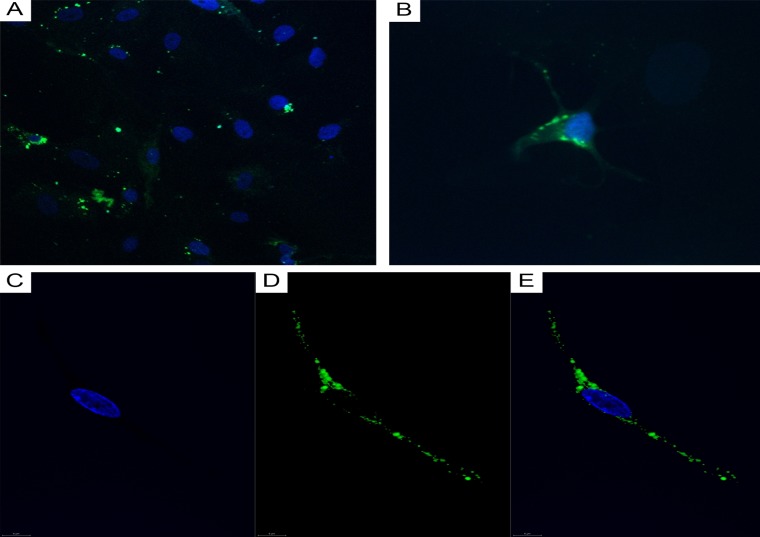
CLSM of glioblastoma and astrocytoma primary cultures. **(A)** Glioblastoma tumor cells (DAPI/nuclei in blue; GFAP/Alexa Fluor488 in green; 200x); **(B)** astrocytoma tumor cells (DAPI/nuclei in blue; GFAP/Alexa Fluor488 in green; 400×). (C–E) CD117+ cells in a glioblastoma culture (60 0×). **(C)** Blue fluorescence of the cell nucleus (DAPI); **(D)** Green CD117/Alexa Fluor488 fluorescence; **(E)** Overlay image (nucleus in blue; CD117 in green).

CLSM of cell cultures isolated from GBMs and astrocytomas revealed GFAP^+^ tumor cells (Figure 9A and 9B) and CD117^+^ cells featuring Tc morphology (Figure 9C–9E).

**Figure 10:**
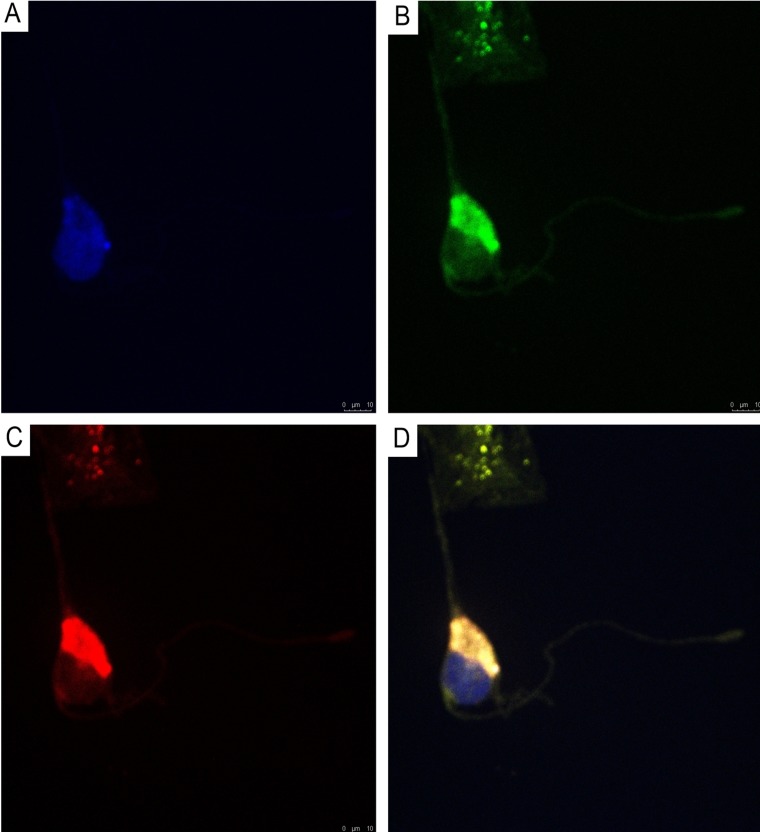
CLSM of astocytoma primary culture. **(A)** Blue fluorescence of cell nuclei (DAPI); **(B)** Green fluorescence of CD34/Alexa Fluor488 on the telopodes and the telocyte cell body; **(C)** Red fluorescence of connexin43/Alexa Fluor568 on the telopodes and the telocyte cell body; **(D)** Overlay of images (A–C). Co-localization (CD34/connexin43) was observed as yellow fluorescence on the telopodes and the telocyte cell body; 400×.

**Figure 11:**
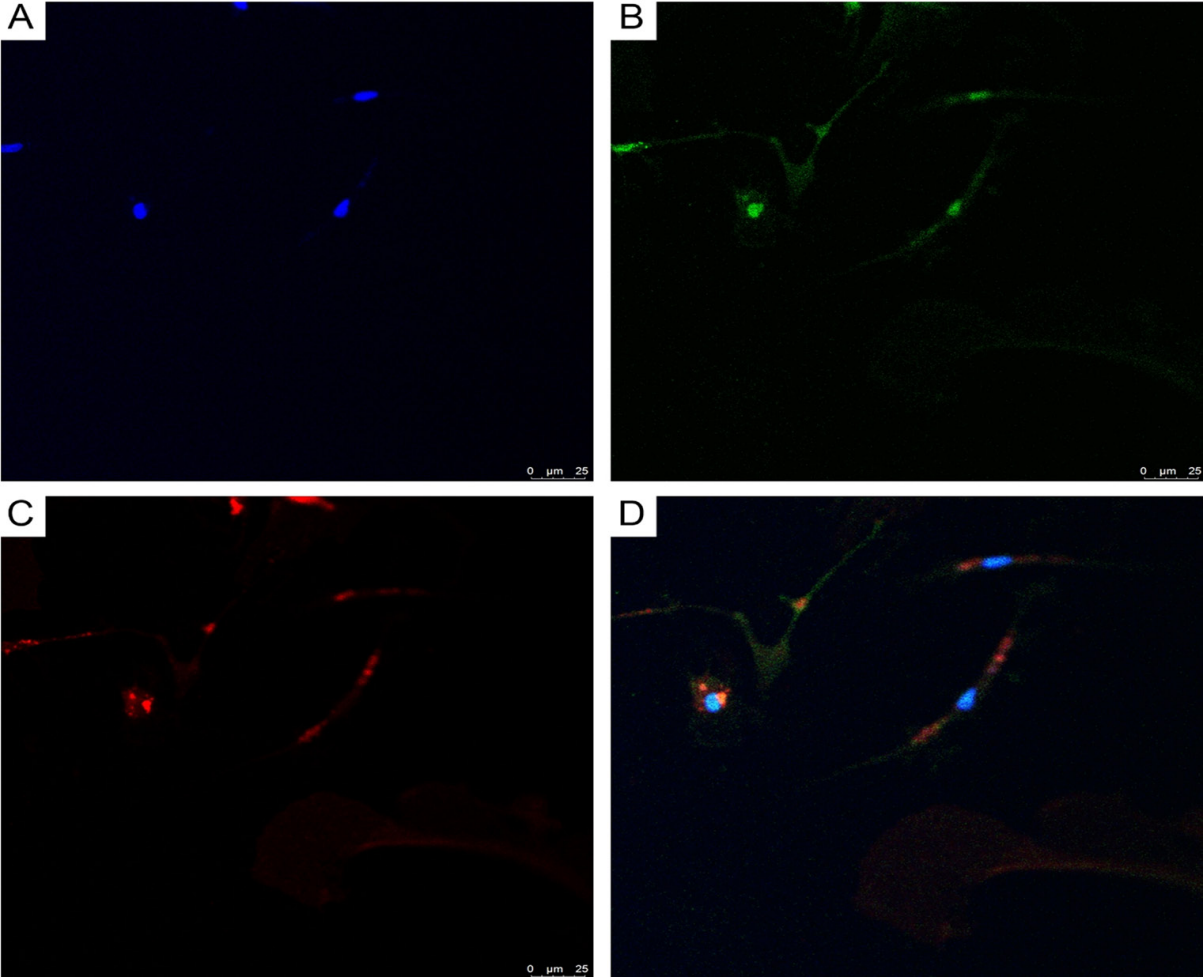
CLSM of glioblastoma primary culture. **(A)** Blue fluorescence of cell nuclei (DAPI); **(B)** Green fluorescence of NeuroD1/Alexa Fluor488 in telocyte nuclei; **(C)** Red fluorescence of connexin43/ Alexa Fluor568 on the telocyte telopodes; **(D)** Overlay of images (A–C) reveals NeuroD1/connexin43 same cell (Tc) co-expression; 200×.

Using double immunofluorescence, we demonstrated CD34/connexin43 co-expression in diffuse astrocytoma culture (Figure 10) and NeuroD1/connexin43 co-expression in GBM culture (Figure 11) in cells with Tcs morphology (featuring long, thin prolongations). CD34/connexin43 co-localization was observed on the telopodes and the cell body as yellow fluorescence (Figure 10D). With NeuroD1/connexin43 double-staining, dual signals from individual (same) Tc cells were observed: NeuroD1 in the nucleus (green fluorescence) and connexin43 in the cytoplasm (red fluorescence) (Figure 11D).

**Figure 12:**
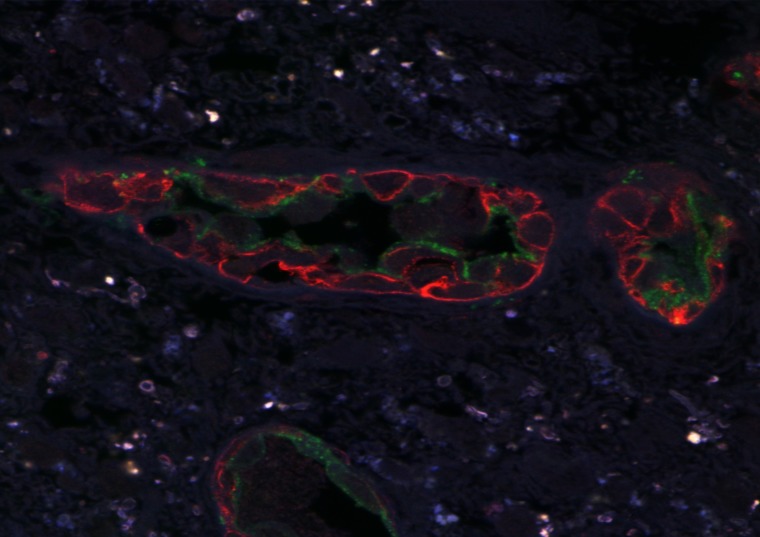
CLSM of glioblastoma. CD34 + /Alexa Fluor488 (green) and NG2 + / Alexa Fluor568 (red) cells are seen in glioblastoma vessels. Paraffin section; 200×.

**Figure 13:**
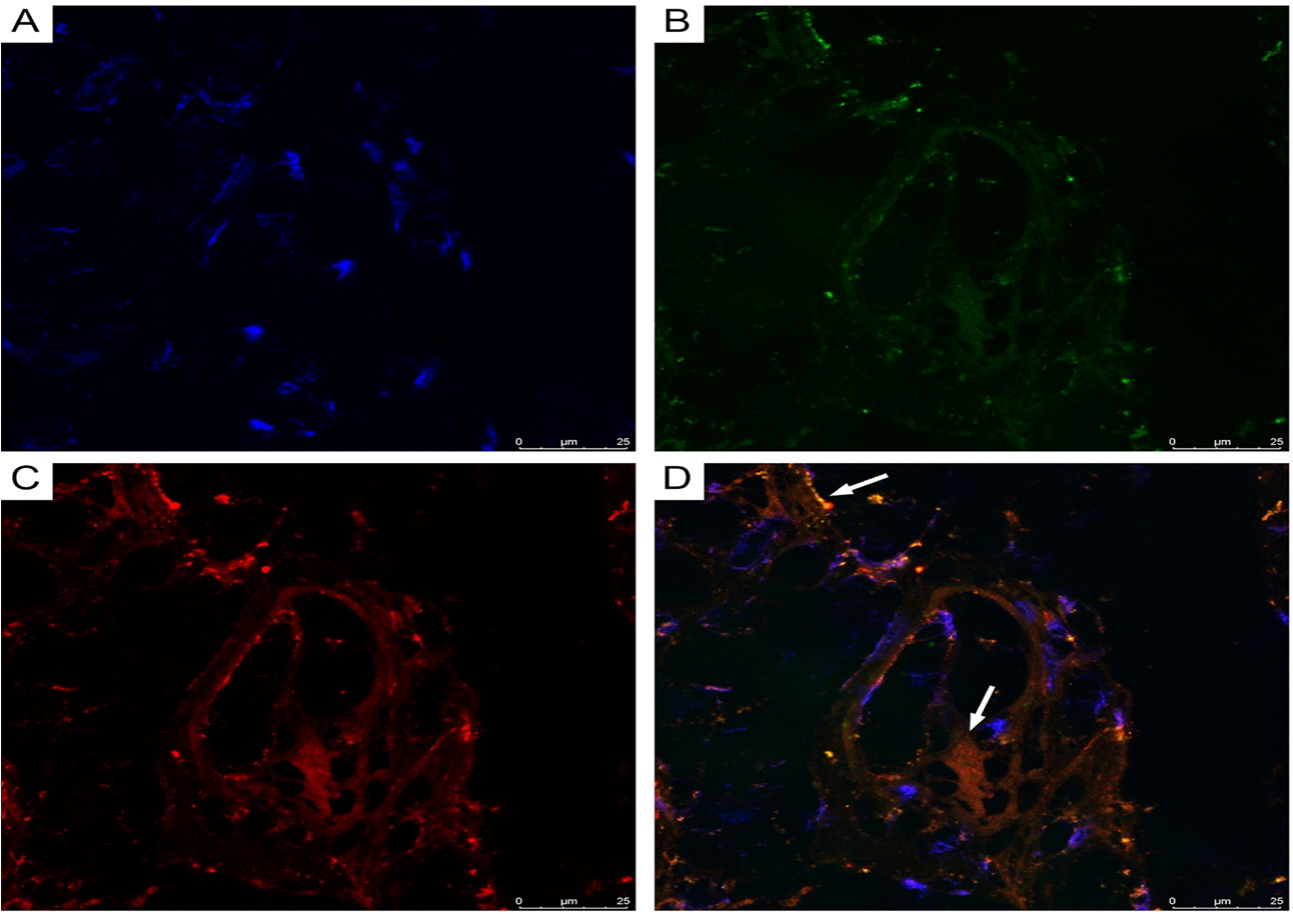
CLSM of glioblastoma. **(A)** Blue fluorescence of cell nuclei (DAPI); **(B)** Green fluorescence of CD34/Alexa Fluor488; **(C)** Red fluorescence of NG2/Alexa Fluor568; **(D)** Overlay of images (A–C). Same-cell CD34/NG2 co-expression (orange fluorescence), indicated by arrows, is visible in glioblastoma vessels (frozen sections, 200×).

**Figure 14:**
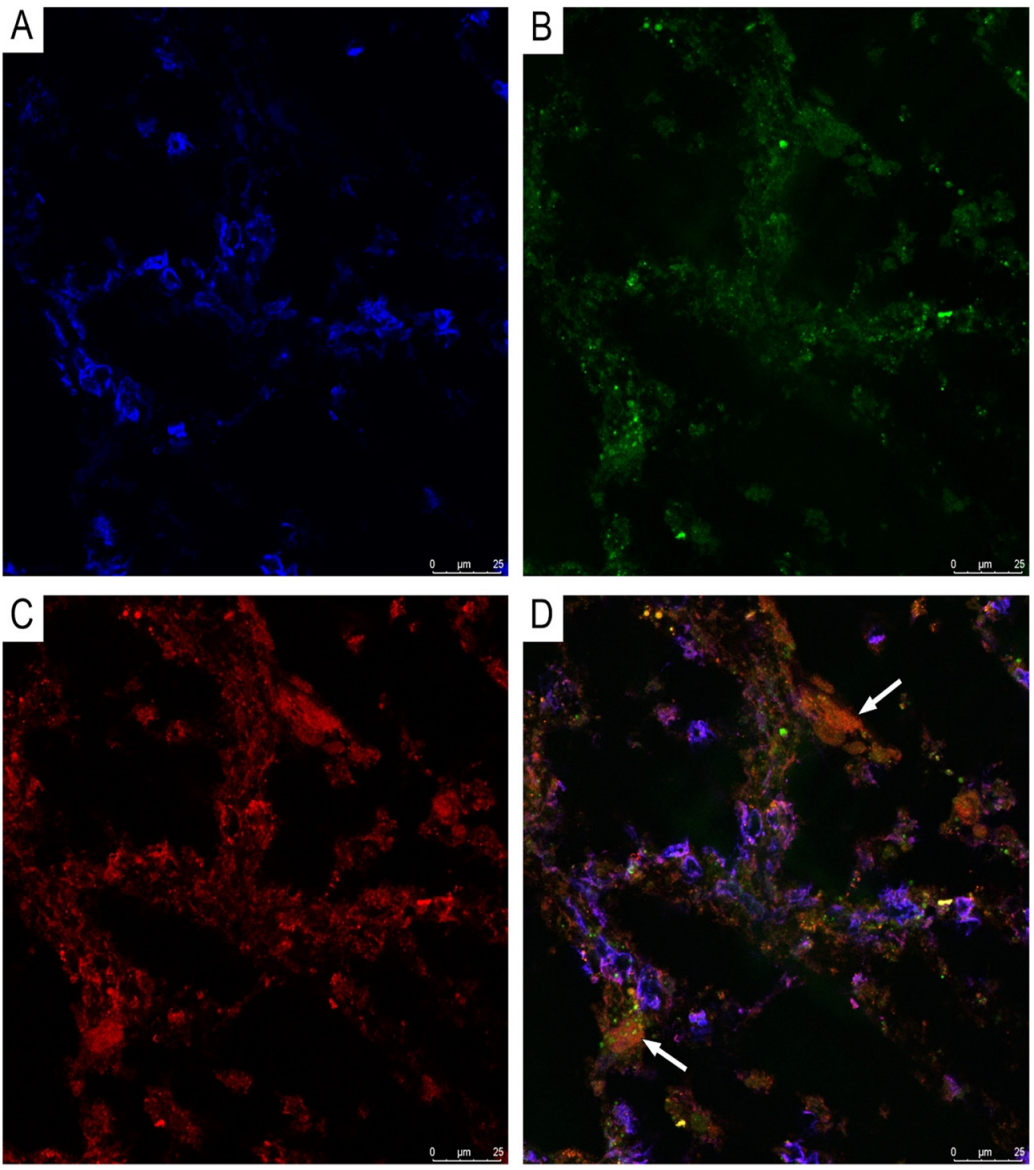
CLSM of glioblastoma. **(A)** Blue fluorescence of cell nuclei (DAPI); **(B)** Green fluorescence of CD13/Alexa Fluor488; **(C)** Red fluorescence of CD117/Alexa Fluor568; **(D)** Same-cell CD117/CD13 co-expression (orange fluorescence), indicated by arrows, is visible in glioblastoma vessels (frozen sections, 200×).

CLSM of paraffinized and frozen GBM sections demonstrated CD34^+^ and NG2^+^ immunophenotype cells in the walls of vessels (Figures 12 and 13). In our view, this can be interpreted as: CD34^+^ endothelial cells, CD34^+^ Tcs, and NG2^+^ Pcs. Cells with CD34/NG2 co-expression (Figures 12 and 13) and CD117/CD13 co-expression were also detected (Figure 14); we interpret these as cells featuring a mixed (Tc/Pc) immunophenotype.

### Transmission electron microscopy

**Figure 15:**
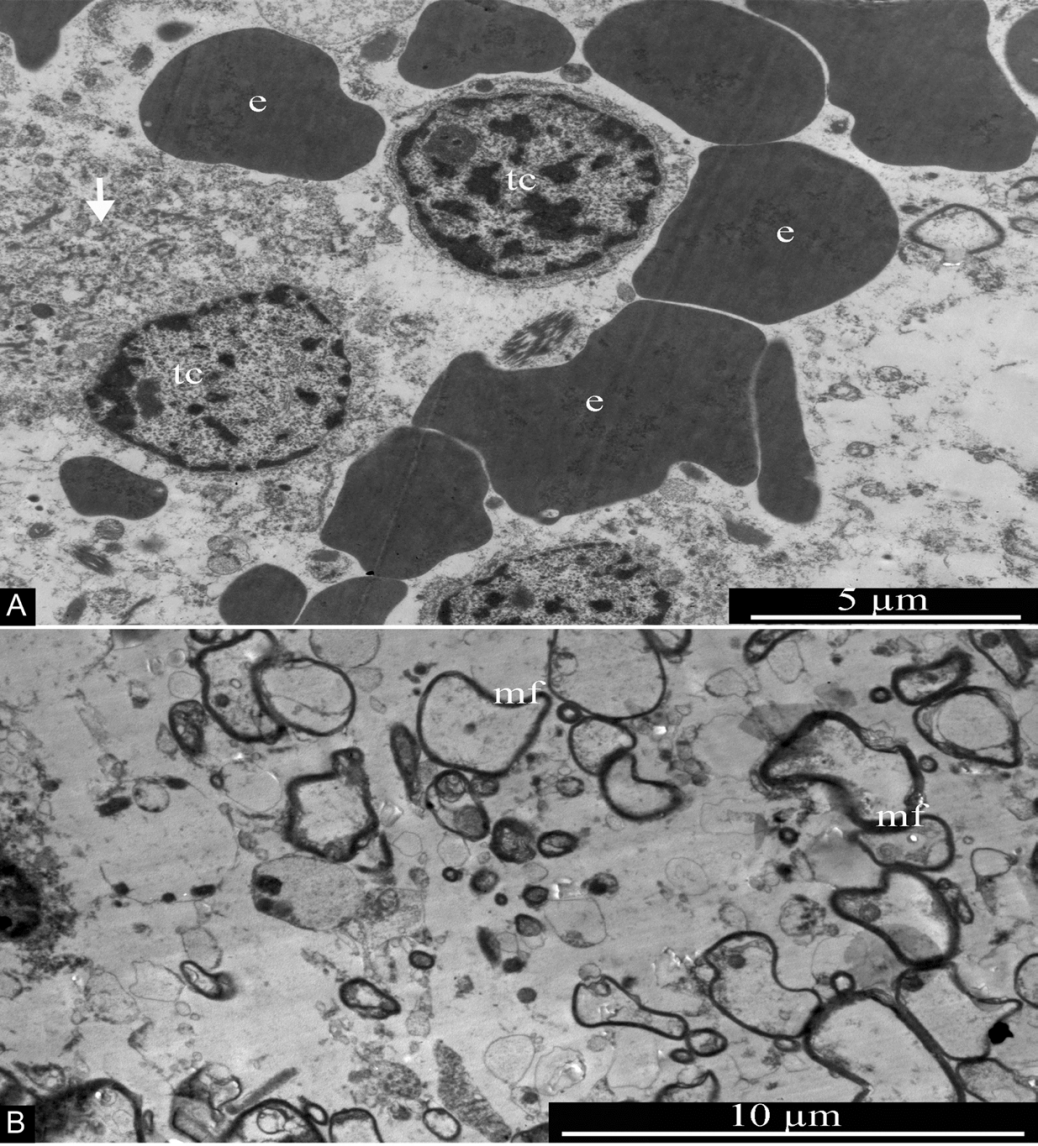
General ultrastructure of glioblastoma. **(A)** Tumor cells with signs of necrotic destruction (arrow). Erythrocytes are seen in the vicinity of tumor cells, without any association with blood vessels. **(B)** Transverse section of multiple myelin fibers, of various sizes, within the glioblastoma. Abbreviations: tc, tumor cells; e, erythrocytes; mf, myelin fibers.

In GBM samples examined by electron microscopy, tumor cells featuring round nuclei about 5 μm in diameter and narrow areas of perinuclear cytoplasm, in many cases without pronounced differentiated organelles, were detected. Transformed cells in necrotic death (featuring disruption of membrane integrity and fragmentation of the cytoplasm) were frequently seen (Figure 15A). Some areas of the GBM samples studied were enriched with osmiophilic myelin fibers of various diameters (Figure 15B). Along with tumor cells and myelin fibers, numerous erythrocytes were present in the samples, often not associated with blood vessels (Figure 15A); this confirms the complete functional destruction of the blood-brain barrier in GBMs.

**Figure 16:**
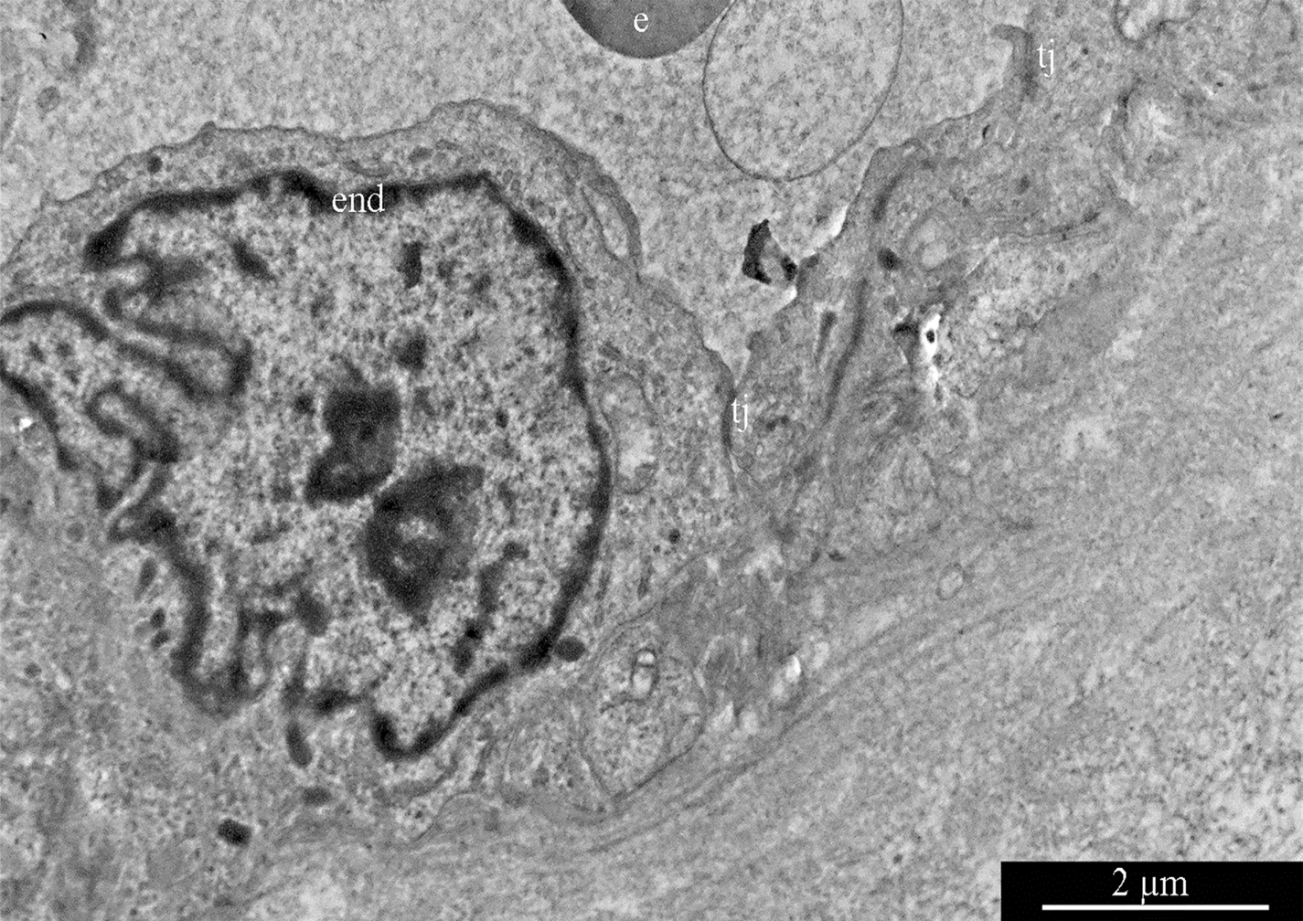
Glioblastoma blood capillary. Abbreviations: end, endotheliocyte; tj, tight junction; e, erythrocyte.

GBM blood capillaries are formed by thin endothelial cells connected by tight contacts. The thickness of the endothelium in the peripheral region of endotheliocytes is 0.4-0.6 μm. The central, nucleus-containing region has a thickness of 3-5 μm. Endotheliocyte nuclei are generally rounded, but they can form deep, uneven invaginations of the nuclear envelope (Figure 16).

**Figure 17:**
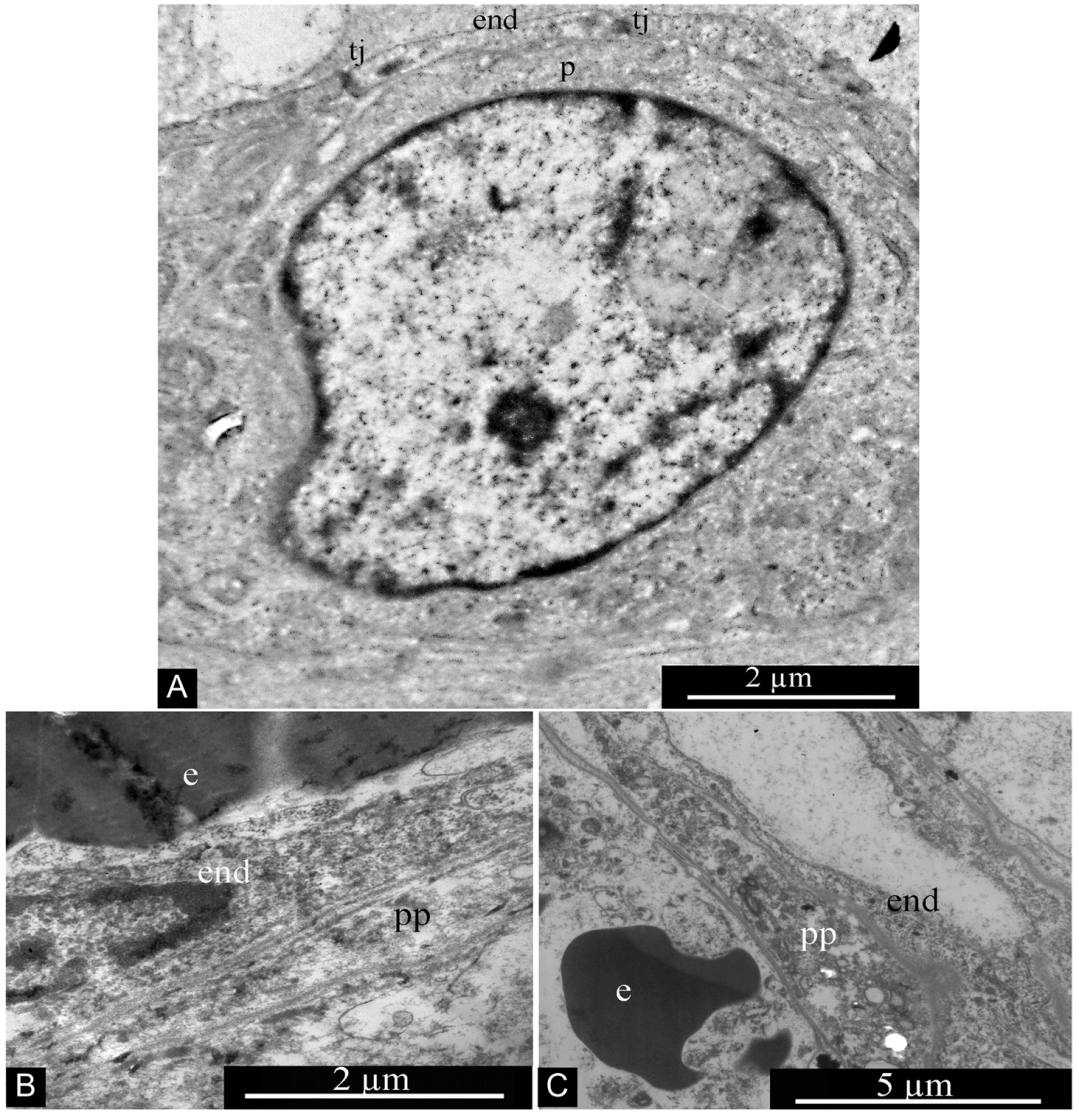
Pericytes in glioblastoma blood capillary. **(A)** Pericyte associated with wall of glioblastoma blood capillary. **(B and C)** Pericyte processes in glioblastoma blood capillaries. Abbreviations: p, pericyte; end, endotheliocyte; tj, tight junction; e, erythrocyte; pp, pericyte process.

Two interstitial cell types are associated with GBM capillaries: Pcs and Tcs. Pcs are localized directly on the outer surface of the endothelium and are in close contact with endotheliocytes; often the Pc body is not separated from the endothelium by the basement membrane (Figure 17A). Pc peripheral processes are quite massive, featuring a thickness of 1–2 microns without evident thickenings or narrowings (Figure 17B and 17C).

**Figure 18:**
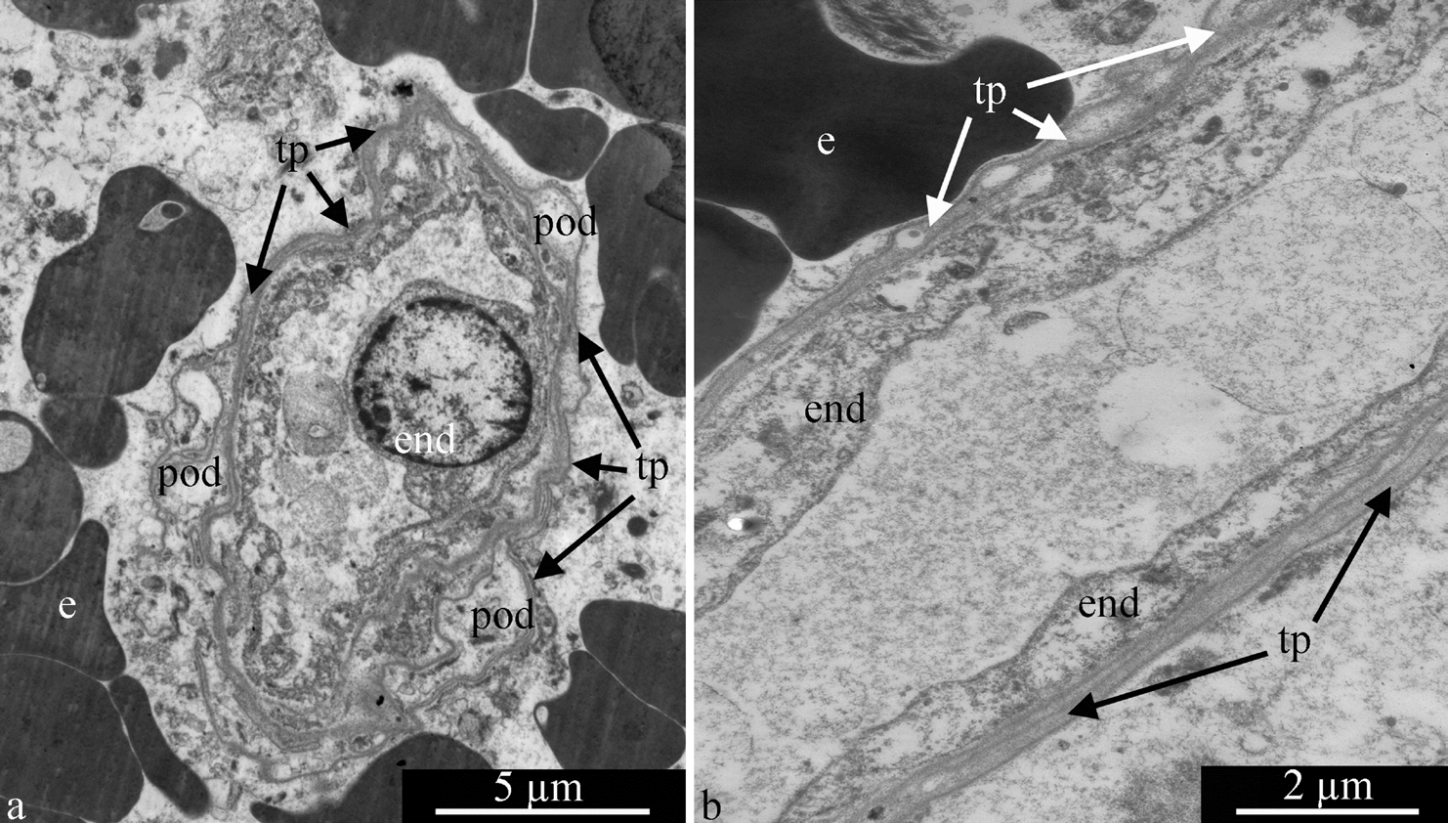
Processes of telocytes (telopodes) in glioblastoma ’s blood capillaries. Telopodes are visible in **(A and B)**. Alteration of dilated podomes and thin podomeres is visible in (A). Erythrocytes are located outside the capillaries in (A and B). Abbreviations: end, endotheliocyte; e, erythrocyte; tp, telopode; pod, podome.

In contrast, Tcs form much longer and thinner processes, telopodia, which are 0.1–0.2 μm thick (Figure 18A and 18B). The telopodia ultrastructure seen in glioblastomas is fully consistent with that previously described repeatedly in a number of tissues and organs. Characteristic dilations are seen, the podoms, which alternate with thin segments termed podomeres (Figure 18A).

Thus, a number of methods converge to show the presence of Tcs in tumors. IHC, electron microscopy, and CLSM reveal them in GBMs. Cultures derived from GBMs and astrocytomas, examined by phase-contrast and CLSM, also show the presence of Tcs. The number of Tcs in GBM specimens was 10 times more than in astrocytoma. In normal gray and white matter, Tcs and NG2^+^ Pcs constitute less than 0.5% of the population. Four cell immunophenotypes were found in GBM vessels: endotheliocyte; Pc; Tc; and a mixed Pc/Tc type.

## DISCUSSION

Based on numerous descriptions of Tc function [58, 59], the presence of Tcs in GBM vessels, as seen by IHC and confocal microscopy, is not entirely surprising. Here, we have proven the presence of Tcs in GBM and astrocytoma primary cultures. Our immunohistochemical study with CD117 revealed the presence of Tcs in tumor vessel walls and in glial scars; some authors have described Pcs in these locations [60]. Svensson et al. [61] confirmed that activated Pcs infiltrating glioma were mainly localized to the tumor vessel wall. Cheng et al. [62] believe that glioma stem cells often reside in perivascular niches and that they may undergo mensenchymal differentiation; the authors showed that glioma stem cells give rise to Pcs most likely in order to support vessel function and tumor growth.

The Pc immunophenotype differs from the Tc type by the absence of expression of several markers: CD117 (c-Kit); CD34; S100; NSE; and connexin43. In our study, GBM vascular cells expressed CD117. Tcs from glioma culture showed CD34/connexin43 co-expression in addition to CD117 expression. This is fully consistent with the immunophenotype of interstitial pacemaker cells.

Moreover, by using CLSM microscopy to study Tc cultures isolated from gliomas, we proved that these cells express Neuro D1. Using GBM paraffin sections, we also revealed, by IHC, the expression of this transcription factor in the nuclei of vascular wall cells. In recent decades, Neuro D1 has been found in the adult nervous system and in the endocrine cells of primitive neuroectodermal tumors [63]. It is known that this factor is involved in neurogenesis, including control of potential trans-differentiation, in which other cell types may become neurons; it is also a general regulator of brain development [64].

Neuro D1 is known to play an important role in the differentiation, morphogenesis, and maintenance of cells of the central nervous system [65]. This transcription factor is expressed in the pituitary gland and in the progenitor cells of the “endocrine” part of the pancreas, both during embryogenesis and later [66, 67]; it is also expressed in neuroectoderm cells [68]. In our work, it was also found in GBM tumor cells.

Adult neurogenesis is the process of generating new, functional neurons from neural stem cells and neural progenitor cells in order to react to and adapt to additional stimuli in various physiological and pathological conditions. These cells are predominantly located in the SVZ, and they can migrate and differentiate into new neurons [69]. They can also be activated in the meninges or the choroid plexus, where Tcs have been described [70, 71]. In addition, our immunohistochemical study of GBM revealed that, in vessel walls, there are cells expressing not only CD117, but also NG2 with SMA. Dual-label IHC demonstrated the presence of cells with NG2/SMA co-expression, i.e. Pcs. CLSM of GBM revealed cells with co-expression patterns such as CD13/CD117 and NG2/CD34. In other words, cells with mixed Pc/Tc immunophenotypes were seen. In vascular walls, we also observed a number of cells without CD34/NG2 co-expression (CD34^+^ endothelial cells, CD34^+^ Tcs, and NG2^+^ Pcs). Under malignant tumor conditions, stromal cells may change their immunophenotype, as reported by Díaz-Flores et al. [72], and Erdag et al. [73]. They have described CD34^+^/SMA^+^ cells in the peripheral areas of scars.

In humans, isolated Pcs expressing CD146, NG2, PDGFRb, and SMA have been theorized to be a native source of mesenchymal stem/stromal cells (MSC) [74]. Apparently, the fifth described mechanism of glioma neovascularization involves the trans-differentiation of glioma cells into 3 immunophenotypes: (1) CD117^+^/CD34^+^/connexin43^+^/NeuroD1^+^ Tc immunophenotype; (2) SMA^+^/NG2^+^ Pc immunophenotype; and (3) a mixed, transitional Tc/Pc immunophenotype with CD13/CD117 and NG2/CD34 co-expression. It is known that GBM vessels are functionally and morphologically abnormal. This phenomenon ensures the maintenance and progression of the tumor. A so-called “vascular or perivascular niche” concept of GBM, in which tumor areas are potentially invulnerable due to shielding from chemotherapy, has been highlighted [75].

Vascular “strengthening” in GBM is confirmed, in our work, by the fact that there are 10 times more CD117^+^ Tcs in this tumor than in astrocytomas. The number of these CD117^+^ Tcs has a significant linear correlation with the proliferative activity index of tumor cells. Moreover, in the gray and white matter of control group (tumor^–^) specimens, we observed only single CD117^+^ Tcs in the vascular walls. Accordingly, MSC have been proposed to arise from perivascular cells termed Pcs [76]. It has been suggested that MSC-like cells originate from Pcs that have become activated after tissue damage [77]. The concept of MSC is being revised [78] due to the large number of studies that refute this fact, and Pcs are being proposed for the role of this “intriguing” cell.

Our study shows that the Tc immunophenotype in gliomas is characterized by NeuroD1 expression. The presence of new Tc immunophenotypic characteristics in gliomas aligns with the opinions of Cretoiu et al. [79] and Diaz-Flores et al. [80], who indicate that Tcs change phenotype according to organ. From our point of view, the presence of Neuro D1 in glioma Tcs confirms the assumption of Popescu et al. [14] that Tcs can participate in neurogenesis and regulate the development of the brain as a whole. It is possible that Tcs are derived from Pcs. In any case, we found cells with mixed, transient immunophenotypes (with CD13/CD117, NG2/CD34 co-expression) which can be attributed to NG2-glia. It’s entirely possible that researchers may, at times, be interpreting one type as various cell types, in the absence of detailed immunophenotype analysis.

## MATERIALS AND METHODS

### Clinical samples

Fifteen hemispheric Grade IV GBMs (gliob^+^ study group), 10 hemispheric Grade II diffuse astrocytomas (astro^+^ comparison group), and 5 normal, tumor-free brains (control group) were studied. Hemisphere gliomas were removed, as follows: 3 GBMs with primitive neuronal component; 2 giant cell GBMs, 10 small cell GBMs, and 10 diffuse astrocytomas. Tumor-free frontal lobes (control group) were obtained from deceased cardiovascular disease patients; specimens were taken within 4 hours after death. GBM^+^ group patient ages ranged from 48 to 76 years (62.0±7.1 yrs av.), and the group consisted of 13 women and 2 men. Astro^+^ group patient ages ranged from 35 to 71 years (54.0±12.4 yrs av.), and the group consisted of 6 women and 4 men. The control group (tumor-free brains) was comprised of 2 women and 3 men; the mean age was 55±3 years, and the ages ranged from 40 to 68 years old. Histological study featured hematoxylin and eosin staining. Table 1 summarizes patient clinical characteristics and the specimen analysis methods used.

### Antibodies

A detailed list of antibody reagents used for immunohistochemistry and confocal laser scanning microscopy (CLSM), including their dilutions, is provided in the Supplementary Materials.

### Immunohistochemistry

Histological and immunohistochemical studies were carried out on sectioned paraffin block embedded samples. IHC study was performed using the peroxidase-based detection method. Immunohistochemistry (IHC) with antibodies to GFAP, Ki-67, CD117, NeuroD1, CD34 and NG2 was performed on gliomas. Double immunohistochemical staining (NG2/SMA) was performed in 6 GBM cases (patients 6–11, Table 1). A CD34/CD117 cocktail was used in 2 astrocytoma cases (patients 16 and 17, Table 1). An immunohistochemical study was also performed on normal brain samples with antibodies to CD117, NeuroD1, and NG2 (patients 26–30, Table 1). Taking into account the high importance of CD117 as a Tc marker, we used three CD117 antibodies: mouse monoclonal c-Kit (clone 1657, Novusbio, USA); rabbit monoclonal c-Kit (clone AH26, Genemed, San Francisco, CA, USA); and rabbit polyclonal c-Kit (Diagnostic BioSystems, Netherlands). In order to confirm the specificity of the CD117, Neuro D1, and NG2 antibodies, we performed IHC staining of skeletal muscle sections with these antibodies as negative controls; those staining controls were completely negative (Supplementary Figure 1A–1C, Supplemental Materials). A complete description of the IHC procedure is provided in the Supplementary Materials.

### Primary culture of telocytes from gliomas

Tumor fragments were removed under sterile conditions and placed into wide (50 ml) tubes with phosphate-buffered saline (PBS). Following rinsing with fresh PBS to remove blood, tumor samples were minced into millimeter-sized pieces in a sterile culture dish containing a solution of collagenase type II (V900892; Sigma-Aldrich, St. Louis, MO, USA) in PBS, followed by incubation at 37°C for 30 min. Collagenase was then deactivated by Dulbecco's Modified Eagle Medium (DMEM) (12400-024, Gibco; Thermo Fisher Scientific, Inc., Waltham, MA, USA) supplemented with 1% glutamine and 1% penicillin/streptomycin (PS). Fragments were centrifuged at 300g for 10 min at room temperature. Supernatants were removed, and sediments were seeded onto sterile culture dishes following re-suspension in fresh DMEM. Samples were cultured (37˚C with CO_2_ and humidity) for 7 days, with replacement of media every 2 days. Cell cultures were examined daily using an inverted microscope. After 7 days, colonies of cells had begun to form around the small explants. By 2 weeks, they covered more than 70% of their dishes. Characterization cell culture Tcs was performed using routine phase contrast microscopy and CLSM.

### Confocal laser scanning microscopy

CLSM of 4 glioma primary cultures (patients 12,13,16, and 17) was performed using an indirect double immunofluorescent staining procedure with primary antibodies recognizing GFAP/CD117, CD34/connexin43, and NeuroD1/connexin43; nuclei were counterstained with DAPI (AppliChem). CLSM of GBM sections, both frozen (patients 4 and 5) and paraffinized (patients 6 and 7), was performed. Double indirect immunofluorescent staining of sections with CD34/NG2 and CD13/CD117 primary antibodies combinations was performed. *Alexa Fluor_488_ goat anti-mouse* and *Alexa Fluor_568_ goat anti-rabbit IgG* were used for secondary antibody labeling. For all of the specimens listed above, CLSM was performed using a TCS SP8 microscope (Leica, Germany) equipped with a 405 nm diode laser (for DAPI excitation), a 488 nm Argon laser (for Alexa Fluor_488_ exc.), and a 561 nm DPSS laser (for Alexa Fluor_568_ exc.). The CLSM method is further detailed in the Supplementary Materials.

### Electron microscopy

Each tumor specimen was cut into small pieces, approximately 1–2 mm^3^ in size, and pre-fixed with 2.5% glutaraldehyde in PBS (pH 7.4) for 45 min at room temp. These pieces were washed three times with PBS and post-fixed 1% PBS-buffered OsO_4_ for 1 hour. Specimens were then dehydrated in a series of ethanol solutions of gradually increasing concentration and embedded in Epon epoxy resin. Ultra-thin sections (70–90 nm) were obtained using a Leica EM UC7 ultramicrotome. Sections were transferred onto copper grids, stained (uranyl acetate and lead citrate), and examined using a JEM 1011 TEM (JEOL, Japan) equipped with a Morada digital camera (Olympus, Japan).

### Morphometry and statistics

Morphometric analysis was performed using an automated image analyzer (Image Scope Color M, Russia). In order to analyze the relative quantities of cells expressing select antigens, 10 high-power fields (400× magnification) were evaluated per specimen. For CD117 (patients 1–10, 16–30) and Ki-67 (patients 1–25), percentages of the average number of expressing cells, in relation to overall cells, were separately calculated. Statistical analysis of the acquired data was done using Statistica v.10 software (StatSoft, Russia). For normal distributions, the significance of differences in quantitative characteristics was interpreted using the Student’s t-test. For other types of distribution, we used non-parametric methods of analysis, namely the Mann–Whitney test for independent samples and the Wilcoxon test. Differences between groups were defined as significant when *p* <0.05. In order to evaluate the correlation of two variables, we applied Spearman rank correlation analysis. Correlation coefficient (r) interpretation: r <0.3 as weak association; r=0.3–0.5 as moderate; r= 0.5–0.7 as significant; r=0.7–0.9 as strong; and r>0.9 as very strong. Correlation was considered as positive if r>0 and negative if r<0.

## CONCLUSIONS

In this study, a number of methods (IHC, CLSM, electron microscopy) were used to analyze glioma vessels and tumor-derived cultures. Tcs featuring a CD117^+^/CD34^+^/connexin43^+^/NeuroD1^+^ immunophenotype were seen. In GBM vessels, four immunophenotypes were found. These correspond to endotheliocytes, Pcs, Tcs, and a mixed telocyte/pericyte immunophenotype. Further refinement of targeted therapies and development of new cellular therapies will require improved understanding of biology, at the molecular and histological levels, in several areas such as: the origin and function of Tcs; their relationship with Pcs; and the roles of both Tcs and Pcs in the oncogenesis of brain tumors. The authors hope that the analysis presented here advances our understanding in those directions.

## SUPPLEMENTARY MATERIALS FIGURES



## Abbreviations

3D: 3-Dimensional
Ab: Antibody
ANG-2: Angiopoietin-2
CD: Cluster of Differentiation
CLSM: Confocal Laser Scanning Microscopy
DMEM: Dulbecco’s Modified Eagle Medium
DSIHC: double stain immunohistochemistry
EGF: Epidermal Growth Factor
EM: electron microscopy
FGF: Fibroblast Growth Factor
GBM: Glioblastoma multiforme
GFAP: Glial Fibrillary Acidic Protein
H: Histology
IDH: Isocitrate Dehydrogenase
IHC: Immunohistochemistry
MA: Morphometric Analysis
MRI: Magnetic Resonance Imaging
MSC: Mesenchymal Stem Cells
NG2: Neuron-Glial Antigen 2
NOS: Not Otherwise Specified
NSE: Neuron-Specific Enolase
PBS: phosphate-buffered saline
PC: primary culture of telocytes from gliomas
Pcs: Pericytes
PDGFR: Platelet-Derived Growth Factor Receptors
PS: Penicillin/Streptomycin
SMA: Smooth Muscle Actin
TGFL: Transforming Growth Factor
Tcs: Telocytes
VEGF: Vascular endothelial growth factor.
