# Supplementary material for: Morphological and immunophenotypic characterization of perivascular interstitial cells in human glioma: Telocytes, pericytes, and mixed immunophenotypes
Source: Oncotarget. 2020 Jan 28;11(4):322–46. doi: 10.18632/oncotarget.27340 (PMC6996916; doi:10.18632/oncotarget.27340)
Supplement: Supplementary file 1 [file oncotarget-11-322-s001.pdf]

Morphological and immunophenotypic characterization of perivascular interstitial cells in human glioma: telocytes, pericytes, and mixed immunophenotypes

SUPPLEMENTARY MATERIALS

IMMUNOHISTOCHEMICAL STUDY

For study by IHC (see Table1), specimen paraffin sections were de-paraffinized with xylene and re-hydrated. For endogenous peroxidase inactivation, sections were treated with 3% hydrogen peroxide (5 min. at room temperature, hereafter "r.t.") and then washed in distilled water. For antigen retrieval, slides with sections were boiled in EDTA (pH 9.0) in a water bath (Thermo Fisher). Then, sections were washed in Tris-Buffer with Tween 20 (2 washes of 5 min. each). Bovine serum incubation was carried out, for 20 min. at r.t., in a container with wet filter paper. Next, sections were incubated with primary antibody (Ab) at r.t. for 30 min. Here, we used: anti-GFAP and anti-NeuroD1 mouse monoclonal Abs; and anti-CD117 and anti-NG2 rabbit polyclonal Abs. Secondary Ab incubations were for 30 min. (r.t.) and used the corresponding mouse or rabbit EnVision™+ Peroxidase System (DAKO, Denmark). Next, sections were washed twice in Tris-Buffer with Tween 20. Coloured DAB reaction products were visualized directly by light microscopy. After washing in distilled water, the sections were counterstained with hematoxylin for 2 minutes, dehydrated, and then mounted using a permanent mounting medium (Polystyrol, BioMount, Italy).

For double immunostaining, the de-paraffinized and re-hydrated sections were treated with Tris-EDTA (pH 9.0) at 95-98°C in a water bath for 35 minutes and allowed to cool at r.t. for 20 min. In order to inactivate endogenous peroxidase, sections were treated with 3% hydrogen peroxide (5 minutes at r.t.) and washed in distilled water. Tissue specimens were then incubated with UltraVBlock for 10 minutes (r.t.) to diminish non-specific background staining. Primary Ab cocktail №1 contained mouse anti-SMA Ab and rabbit anti-NG2 Ab. Primary Ab cocktail №2 contained mouse anti-CD34 Ab and rabbit anti-CD117 Ab. Sections were incubated in it for 30 min. Sections were next washed and incubated with secondary Ab cocktail (MultiVision Polymer Cocktail, Thermo Scientific, UK), which contains anti-mouse/AP and anti-rabbit/HRP. Next, LVBlue and LVRed working solutions were applied, and the sections were incubated for 10 min. in each reagent. Finally, the sections were washed, dried, and embedded in permanent mounting medium (Polystyrol, BioMount, Italy). In result, SMA and CD34 was visualized with blue color, and NG2 and CD117 was visualized with red color.

CONFOCAL LASER SCANNING MICROSCOPY

CLSM of cultured Tcs was performed using single or double immunofluorescence. For CLSM of cultured Tcs, the cells of four glioma primary cultures (patients 12, 13, 16, 17; Table 1), after reaching 80% confluence, were detached using 0.25% trypsin/EDTA (Invitrogen; Thermo Fisher Scientific, Inc.) for 5 min. and re-plated on glass coverslips with fresh DMEM for 2 days. After 2 days, DMEM was removed, and cells were washed 3 times in PBS, fixed in 4% formaldehyde (15 min.), and washed 3 more times in PBS. Cells were then permeabilized with 0.1% Triton™ X-100 for 10 min. and blocked with 1% BSA for 1 hour. Next, cells were labeled with mouse monoclonal primary Ab (CD117, NeuroD1, CD34, or GFAP) for 1 hour at r.t. When preparing double immunofluorescence, after additional washing, cells on coverslips were incubated with an additional rabbit polyclonal primary Ab (connexin43) for 1 hour at r.t. Alexa $Fluor_{488}$ goat anti-mouse and Alexa $Fluor_{568}$ goat anti-rabbit secondary Abs were used for visualization (1 hour incubation). After washing, nuclei were counterstained with DAPI (AppliChem). Dako Mounting Medium (DAKO, Denmark) was used for mounting of primary cell culture preparations. In result (confocal visualization): NeuroD1 and CD34 signals were seen as green fluorescence; connexin43 as red; and contrasted nuclei as blue.

In addition, CLSM of GBM specimens using 2 primary Abs mixes (CD34/NG2 and CD13/CD117) was performed on frozen sections (patients 4, 5; Table 1) and paraffin sections (patients 6,7; Table 1). Frozen GBM sections (2-4 μm thick) were cut on a Leica CM1510 S cryostat and put onto polylysine-coated slides. Frozen sections were then thawed and washed with PBS. Paraffin GBM sections (2 μm thick) were de-paraffinized with xylene and re-hydrated. Sections were then treated with

Tris-EDTA (pH 9.0) at 95-98°C in a water bath for 35 min. for epitope retrieval, cooled to r.t. for 20 min., and washed with Tris-Buffer with Tween 20. Then, both frozen and paraffin sections were incubated in 2% buffered BSA solution (30 min. at r.t.) to block non-specific Ab binding. After washing, the first primary mouse monoclonal Ab (CD13 or CD34) was applied for 1 hour at r.t. Then, after additional washing, the sections were incubated with an additional primary rabbit polyclonal (NG2 or CD117) Ab for 1 hour at r.t. Alexa $Fluor_{488}$ goat anti-mouse and Alexa $Fluor_{568}$ goat anti-rabbit secondary Abs (both Thermo Fisher Scientific) were used for visualization (1 hour incubation). After washing, nuclei in the sections were stained with DAPI (AppliChem). Dako Mounting Medium (DAKO, Denmark) was used for mounting of all tissue specimens. In result (confocal visualization): CD34 or CD13 signals were seen as green fluorescence; NG2 or CD117 as red fluorescence; and contrasted nuclei as blue.

ANTIBODIES

The following primary antibodies were used for IHC and CLSM:

mouse monoclonal GFAP Ab, diluted 1:100 (clone 6F2, DAKO, Denmark). mouse monoclonal Ki-67 Ab, diluted 1:200 (clone MIB-1, DAKO Cytomation, Denmark). mouse monoclonal NeuroD1 Ab, diluted 1:1000 (clone ab60704, Abcam, UK). mouse monoclonal CD34 Ab, diluted 1:100 (clone QBEnd-10, DAKO, Denmark). mouse monoclonal SMA Ab, diluted 1:300 (clone OV-TL 12/30, DAKO, Denmark). mouse monoclonal CD13 Ab, RTU (clone 38C12, Thermo Scientific, UK). mouse monoclonal c-Kit (CD117) Ab diluted 1:100 (clone 1657, Novusbio, USA). rabbit monoclonal c-Kit (CD117) Ab, diluted 1:100 (clone AH26, Genemed, San-Francisco, California, USA). rabbit polyclonal c-Kit (CD117) Ab at a dilution of 1:500 (Diagnostic BioSystems, Netherlands).. rabbit polyclonal connexin43 Ab, diluted 1:100 (Cell Signaling, USA). rabbit polyclonal NG2 Ab, diluted 1:700 (Abcam, UK)

The following secondary antibodies were used for immunohistochemistry:

mouse EnVision™+ System, Peroxidase (DAKO, Denmark). rabbit EnVision™+ System, Peroxidase (DAKO, Denmark). MultiVision Polymer Cocktail (Thermo Scientific, UK)

The following secondary antibodies were used for confocal microscopy:

Alexa $Fluor_{488}$ goat anti-mouse, diluted 1:250 (Thermo Fisher Scientific). Alexa $Fluor_{568}$ goat anti-rabbit, diluted 1:300 (Thermo Fisher Scientific)

CD117 ANTIBODIES USED IN FIGURES

Figure 2

A (c-Kit, Genemed, San-Francisco, California, USA);

B (c-Kit, clone AH26, Diagnostic BioSystems, Netherlands)

C (c-Kit, Genemed, San-Francisco, California, USA);

D (c-Kit, clone AH26, Diagnostic BioSystems, Netherlands)

Figure 5 C (c-Kit, Genemed, San-Francisco, California, USA);

Figure 5 D (rabbit polyclonal Ab, Diagnostic BioSystems, Netherlands);

Figure 9D (mouse monoclonal Ab, clone 1657, Novusbio, USA).

Figure 14C (rabbit polyclonal Ab, Diagnostic BioSystems, Netherlands).

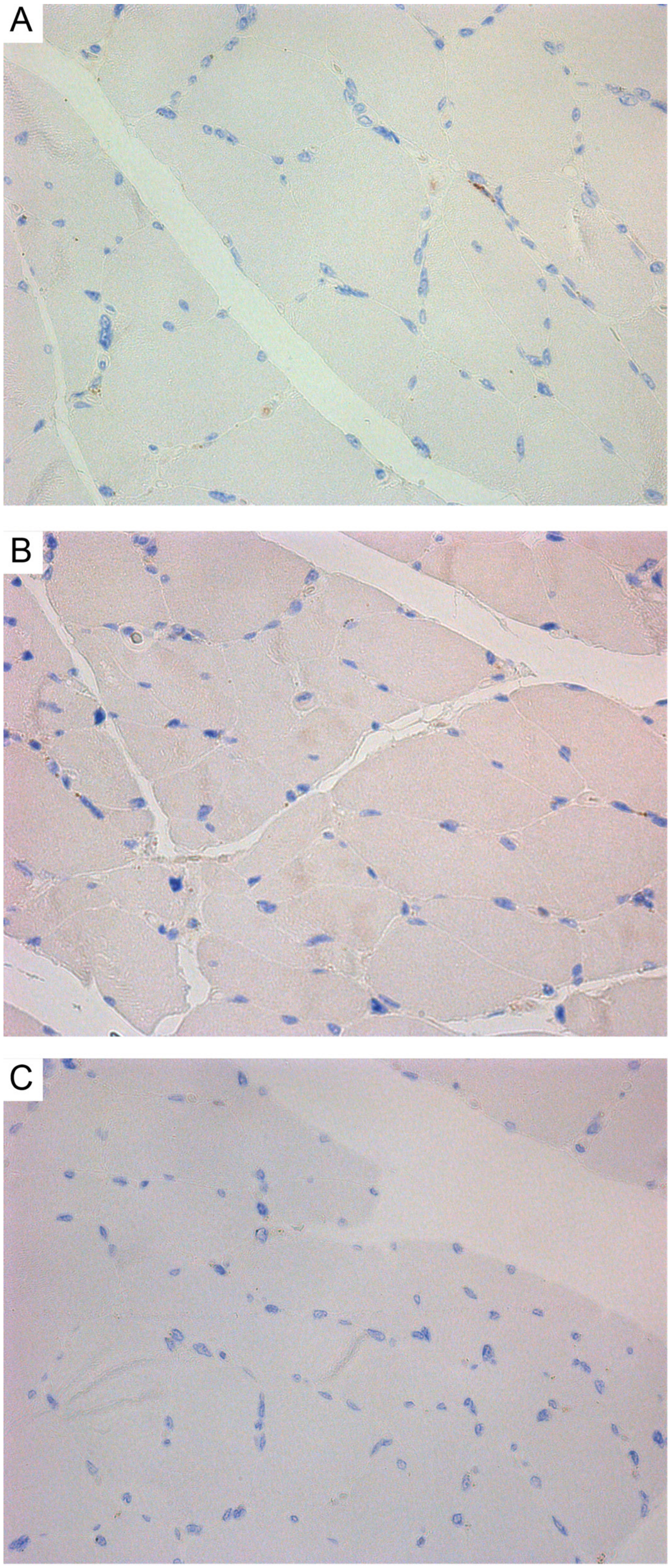

Supplementary Figure 1: Skeletal muscle. (A) expression of CD117 by a single telocyte; **(B)** absence of NG2 expression; **(C)** absence of NeuroD1 expression. 200x.
